# Genome-wide analysis of polymorphism × sodium interaction effect on blood pressure identifies a novel 3′-*BCL11B* gene desert locus

**DOI:** 10.1038/s41598-018-32074-1

**Published:** 2018-09-21

**Authors:** Tsuyoshi Hachiya, Akira Narita, Hideki Ohmomo, Yoichi Sutoh, Shohei Komaki, Kozo Tanno, Mamoru Satoh, Kiyomi Sakata, Jiro Hitomi, Motoyuki Nakamura, Kuniaki Ogasawara, Masayuki Yamamoto, Makoto Sasaki, Atsushi Hozawa, Atsushi Shimizu

**Affiliations:** 1 Division of Biomedical Information Analysis, Iwate Tohoku Medical Megabank Organization, Disaster Reconstruction Center, Iwate Medical University, Shiwa, Japan; 2 Preventive Medicine and Epidemiology, Tohoku Medical Megabank Organization, Tohoku University, Sendai, Japan; 3 Division of Clinical Research and Epidemiology, Iwate Tohoku Medical Megabank Organization, Disaster Reconstruction Center, Iwate Medical University, Shiwa, Japan; 4 Department of Hygiene and Preventive Medicine, School of Medicine, Iwate Medical University, Morioka, Japan; 5 Division of Biomedical Information Analysis, Institute for Biomedical Sciences, Iwate Medical University, Shiwa, Japan; 6 Iwate Tohoku Medical Megabank Organization, Disaster Reconstruction Center, Iwate Medical University, Shiwa, Japan; 7 Department of Anatomy, School of Medicine, Iwate Medical University, Shiwa, Japan; 8 Department of Internal Medicine, School of Medicine, Iwate Medical University, Morioka, Japan; 9 Department of Neurosurgery, School of Medicine, Iwate Medical University, Morioka, Japan; 10 Integrative Genomics, Tohoku Medical Megabank Organization, Tohoku University, Sendai, 980-8573 Japan; 11 Division of Ultrahigh Field MRI, Institute for Biomedical Sciences, Iwate Medical University, Shiwa, Japan

## Abstract

Excessive sodium intake is a global risk factor for hypertension. Sodium effects on blood pressure vary from person to person; hence, high-risk group targeting based on personal genetic information can play a complementary role to ongoing population preventive approaches to reduce sodium consumption. To identify genetic factors that modulate sodium effects on blood pressure, we conducted a population-based genome-wide interaction analysis in 8,768 Japanese subjects, which was >3 times larger than a similar previous study. We tested 7,135,436 polymorphisms in the discovery cohort, and loci that met suggestive significance were further examined in an independent replication cohort. We found that an interaction between a novel 3′-*BCL11B* gene desert locus and daily sodium consumption was significantly associated with systolic blood pressure in both discovery and replication cohorts under the recessive model. Further statistical analysis of rs8022678, the sentinel variant of the 3′-*BCL11B* gene desert locus, showed that differences in mean systolic blood pressure between high and low sodium consumption subgroups were 5.9 mm Hg (*P* = 8.8 × 10^−12^) in rs8022678 A carriers and −0.3 mm Hg (*P* = 0.27) in rs8022678 A non-carriers, suggesting that the rs8022678 genotype can classify persons into sodium-sensitive (A carriers) and sodium-insensitive (A non-carriers) subgroups. Our results implied that rs8022678 A carriers may receive a greater benefit from sodium-lowering interventions than non-carriers.

## Introduction

Sodium is a major cation in the extracellular fluid, and its concentration in the body is mostly maintained through renal excretion and reabsorption. Sodium homeostasis is regulated by endocrine hormones, such as aldosterone and natriuretic peptides, and therefore, is tightly linked to the regulation of blood pressure (BP). Epidemiological studies have demonstrated that reducing dietary sodium intake lowers systolic BP (SBP) and diastolic BP (DBP)^1,2^. The World Health Organisation (WHO) recommends sodium consumption of ≤2 g/day (equivalent to ≤87 mmol/day) for both hypertensive and normotensive adults^3^, but most adult populations around the world are consuming much more sodium than recommended^4^.

For preventing hypertension, public health campaigns have been conducted to promote the reduction of population sodium intake levels in several countries^5^. In addition to ongoing efforts to target general population, genetic identification of individuals who could be at a particularly high risk when exposed to sodium overconsumption should play a complementary role because sodium effects on BP exhibit considerable person-to-person variability^6–8^. Familial studies have shown that inter-individual difference in sodium effects on BP can be explained in part by genetic factors with heritability estimates of up to 51%^9,10^. Identifying genetic polymorphisms that influence sodium effects on BP is a key step to enable identification of individuals at such high risk because distinguishing sodium-sensitive from sodium-resistant subjects is difficult to achieve by using only phenotypic measurements^11,12^.

To this end, numerous genetic studies have defined sodium sensitivity trait based on BP responses induced by short-term dietary sodium intake changes^12^. Candidate gene studies showed that polymorphisms in genes encoding components of the renin-angiotensin-aldosterone system, sympathetic nervous system, renal ion transportation, vascular smooth muscle tone regulation, and reactive oxygen species metabolism are associated with sodium sensitivity^12,13^. A recent genome-wide association study (GWAS) further identified eight novel loci associated with sodium sensitivity^14^.

In addition, population-based genetic studies have investigated polymorphism × sodium intake interactions that affect BP levels. Candidate gene studies reported significant interactions between daily sodium intake and genetic variation in genes encoding angiotensinogen (*AGT*)^15^, angiotensin I-converting enzyme (*ACE*)^16^, and cytochrome P-450 3A5 (*CYP3A5*)^17^. Recently, the first genome-wide study of the polymorphism × sodium intake interaction was conducted with a relatively small sample size (~2,650 Chinese subjects)^18^. However, genome-wide interaction analyses generally require large sample size to identify interactions with modest effect sizes with statistical significance^19^. Moreover, the previous genome-wide study assumed only the additive genetic effect, and therefore, might have failed to detect loci with a dominant/recessive genetic effect. To search for novel polymorphism × sodium intake interactions that influence BP, we estimated sodium intake level of the study participants using a spot urine sample and the verified Tanaka’s formula^20^, conducted a population-based genome-wide interaction study of ~8,750 Japanese subjects, which is >3 times larger than the above-mentioned previous study, and analysed additive, dominant, and recessive genetic effects.

## Methods

### Study subjects

As part of the Tohoku Medical Megabank (TMM) Project, TMM Community-Based Cohort Study (TMM CommCohort Study) is a population-based cohort study designed to realise personalised healthcare and medicine. The study design and recruitment methods were previously described^21^. Briefly, TMM CommCohort Study recruited residents of the Iwate and Miyagi Prefectures (the Pacific coast of the Tohoku region of Japan) from May 2013 to March 2016. The participants that were 20 to 75 years of age answered questionnaires about sociodemographic factors, lifestyle habits, and medical history. Physiological, blood, and urine tests were conducted at the time of enrolment. The approval for the study was obtained from the Institutional Review Board of the Iwate Medical University and Tohoku University. All participants gave written informed consent. This study was conducted according to the principles expressed in the Declaration of Helsinki.

### BP and sodium measurements

Based on the guideline of the Ministry of Health, Labour, and Welfare in Japan, BP was measured twice by trained staff members using automatic devices. In some cases, a single measurement was allowed. For subjects with two BP measurements, the average value was used for genetic analyses. For participants taking antihypertensive medications, BP was imputed by adding 10 and 5 mm Hg to SBP and DBP, respectively^18^. We defined hypertensive subjects as those having SBP ≥140 mm Hg, DBP ≥90 mm Hg, or taking antihypertensive medications.

Urinary sodium and creatinine levels were assayed using spot urine collected at baseline survey. Daily sodium intake level was estimated from the urinary sodium and creatinine levels according to the Tanaka’s formula^20^. The formula was developed to estimate 24-h urinary sodium excretion level from spot urine specimens collected at any time using 591 samples of Japanese individuals from the INTERSALT study. The formula includes sodium and creatinine levels from a spot urine sample as explanatory variables and provides an estimate for the gold standard 24-h urinary sodium excretion level derived from the 24-h urine collection. In a validation population, the correlation coefficient between the estimated and measured levels of 24-h urinary sodium excretion was fairly high (*r* = 0.54); and therefore, the Tanaka’s formula is a convenient and accurate method to estimate population sodium intake^20^. The formula has been frequently used in epidemiological studies^22,23^.

### Genotyping and genotype imputation

Participants of the TMM CommCohort Study who were enrolled in 2013 were genotyped using a HumanOmniExpressExome BeadChip Array (Illumina Inc., San Diego, CA, USA)^24,25^. Based on the genotype data, imputation of sex information and identification of close relationship pairs were performed using PLINK software (version 1.90b3.45)^26^. Subjects with inconsistent sex information between genotype and questionnaire, low call rate (<0.99), non-Japanese ancestry, or close relatives (PI_HAT >0.1875) were excluded. Single-nucleotide polymorphisms (SNPs) with a low call rate (<0.95), low Hardy–Weinberg equilibrium exact test *P*-value (<1 × 10^−6^), or low minor allele frequency (MAF; <0.01) were filtered out. After these procedures, 8,840 subjects and 596,877 autosomal SNPs were retained for genotype imputation.

Genotype imputation was performed using SHAPEIT (version 2.r790)^27^ and Minimac3 (version 1.0.11)^28^ software packages with the 1,000 Genomes reference panel (phase 3)^29^. After genotype imputation, variants with low imputation quality (*R*^2^ < 0.8) and low MAF (<0.01) were excluded, and 7,135,436 variants were retained for subsequent analyses.

### Statistical interaction analyses

We excluded subjects whose body mass index (BMI), SBP, DBP, or daily sodium consumption data were not available (*n* = 72). To statistically test the interaction between daily sodium consumption and genetic variants, we fitted linear regression models with and without an interaction term (model 1: BP = β_0_ + β_G_G + β_E_E; model 2: BP = β_0_ + β_G_G + β_E_E + β_GE_ G × E), where G is genotype variable, E indicates daily sodium consumption (mEq/day) variable, β_0_ is the intercept, β_G_ is the coefficient for variable G, β_E_ is the coefficient for variable E, and β_GE_ is the coefficient for the interaction between variables G and E, with the adjustment for population stratification (20 principal components [PCs]), age, sex, and BMI. The significance of the interaction term (β_GE_) was evaluated by the 1 *df* likelihood ratio test^19^. Although we acknowledge that the joint association test of the main and interaction terms (*i*.*e*., 2 *df* likelihood ratio test^19^) has been used in previous genome-wide interaction studies^18^, we used 1 *df* likelihood ratio test because our aim was to identify polymorphism × sodium interactions rather than to search for BP-associated loci taking into account polymorphism × sodium interactions.

In statistical tests, we considered four genetic effects: dosage, additive, dominant, and recessive models. In the dosage model, genotype dosage estimated from genotype imputation was input into variable G. In other three models, the best guess from posterior probabilities for imputed genotypes^30^ were used. Variants with low MAF (<0.01; for dosage and additive models) or low genotype frequency (<0.01; for dominant and recessive models) in the discovery cohort were excluded from genome-wide interaction analyses. The number of variants analysed was 7,104,936 for dosage model, 7,095,077 for additive model, 4,854,745 for dominant model, and 7,132,180 for recessive model. We applied genomic control correction^31^ to genome-wide *P*-values to avoid false positive detections.

We conducted a genome-wide interaction analysis using a cohort of Miyagi subjects (*n* = 4,527). At the discovery stage, *P*_*discovery*_ < 1 × 10^−5^ after genomic control correction was considered as suggestive significance. Interactions achieving suggestive significance were further examined in an independent cohort of Iwate subjects (*n* = 4,241). We previously showed based on PC analysis that genetic distribution of Iwate subjects was slightly different from that of Miyagi subjects^25^. At the replication stage, *P*_*replication*_ < 0.05 and consistent effect direction of the interaction term with the discovery cohort were considered as nominally significant. In the combined analysis, we analysed a pooled cohort of Miyagi and Iwate subjects, and *P*_*combined*_ < 5 × 10^−8^ was considered as genome-wide significance level. In the combined analysis, results were additionally adjusted for study site.

### Power calculation

To estimate the power to detect interactions, we assumed that residuals of age-, sex-, and BMI-adjusted SBP (or DBP) were distributed according to the following genetic model: BP = β_E_E + β_GE_ G × E, where variable E (daily sodium intake) was sampled from a normal distribution with the mean μ_Na_ and standard deviation σ_Na_, and variable G (genotype) was sampled according to assumed frequency of effect allele or genotype (20% or 50%). Model parameters (μ_Na_, σ_Na_, and β_E_) were estimated from the cohort of Miyagi subjects; μ_Na_ = 164.8; σ_Na_ = 37.9; and β_E_ = 0.041426 for SBP and 0.019897 for DBP. β_GE_ was assumed to be 0.5 × β_E_, 1.0 × β_E_, 1.5 × β_E_, or 2.0 × β_E_. We simulated sodium intake and genotype data for 4,527 individuals, performed the 1 *df* test, and recorded whether the interaction term achieved suggestive significance, for each iteration. We repeated 1,000 iterations to estimate the power for each parameter set.

## Results

### Genome-wide interaction analyses

Characteristics of the study populations are shown in Table 1. DBP, BMI, and proportion of subjects taking antihypertensive medication were similar between the discovery and replication cohorts. Age, SBP, daily sodium consumption, and proportion of hypertensive subjects were slightly higher in the replication cohort than in the discovery cohort. The proportion of females was slightly lower in the replication cohort than in the discovery cohort.

**Table 1:** Characteristics of study populations.

	Discovery (Miyagi)	Replication (Iwate)
*N*	4,527	4,241
Female, %	66.4	63.6
Age, year (mean ± SD)	59.0 ± 11.8	62.6 ± 9.8
SBP, mm Hg (mean ± SD)	128.2 ± 19.2	131.5 ± 19.1
DBP, mm Hg (mean ± SD)	76.7 ± 11.9	76.4 ± 11.0
Hypertension, %	38.0	42.1
Antihypertensive medication, %	23.7	23.8
BMI, kg/m^2^ (mean ± SD)	23.6 ± 3.6	23.5 ± 3.5
Daily sodium consumption, mEq/day (mean ± SD)	164.8 ± 37.9	176.6 ± 38.5
Daily sodium consumption, mg/day (mean ± SD)	3,790 ± 872	4,062 ± 886

SBP, systolic blood pressure; DBP, diastolic blood pressure; BMI, body mass index; SD, standard deviation.

The statistical power to detect interactions with modest effect size (β_GE_ = 2.0 × β_E_; effect allele/genotype frequency = 20%) was estimated at 99.5% (under additive model) and 67.5% (under dominant/recessive model) for SBP and 81.0% (under additive model) and 23.5% (under dominant/recessive model) for DBP. Statistical power values for different parameter settings are presented in Supplementary Table S1.

Genome-wide interaction analyses showed slight departures of observed test statistics distribution from the expectation, *i*.*e*., genomic inflation factors ranged from 1.06 to 1.10 (Supplementary Figs S1 and S2). The inflation factors were hardly changed by varying the number of PCs used in interaction models (Supplementary Table S2). Thus, we applied genomic control correction^31^ to avoid false positive detections (Supplementary Figs S3 and S4). After the correction, the dosage, additive, dominant, and recessive models for SBP found 11, 9, 13, and 13 independent loci, respectively, with the suggestive significance (*P*_*discovery*_ < 1 × 10^−5^; Supplementary Tables S3–S6 and Supplementary Fig. S5). For DBP, 10 (dosage), 14 (additive), 13 (dominant), and 9 (recessive) loci were detected (Supplementary Tables S7–S10 and Figure S6).

Interactions discovered by genome-wide analyses were further examined in the replication cohort. A 14q32.2 locus (sentinel variant was rs8022678) × sodium intake interaction, which was detected by the recessive model for SBP, was significant in the replication analysis (*P*_*replication*_ < 0.05). The effect direction was consistent, and effect size estimates for the interaction term were similar in the discovery and replication cohorts (Table 2). In the combined analysis, the rs8022678 × sodium interaction met the genome-wide significance criterion (*P*_*combined*_ < 5 × 10^−8^). Other interactions detected by genome-wide analyses were not significant in the replication cohort or had inconsistent effect directions between the discovery and replication cohorts (Supplementary Tables S3–S10).

**Table 2:** Significant polymorphism × sodium interaction influencing SBP.

SNP	Chr	Position^*^	Rsq^†^	CG	OG	Population	CGF	β_GE_	SE (β_GE_)	*P* ^‡^
rs8022678	14	99,366,690	0.991	GG	AG/AA	Discovery	0.520	−0.064	0.013	4.7 × 10^−6^
						Replication	0.549	−0.049	0.014	4.1 × 10^−4^
						Combined	0.534	−0.056	0.010	4.3 × 10^−9^

*Chromosomal position was according to GRCh37/hg19 assembly.

^†^Imputation quality in terms of R-square was estimated by Minimac3 software version 1.0.11.

^‡^*P*-value for the discovery cohort was corrected by genomic control method.

SBP, systolic blood pressure; Chr, chromosome; CG, coded genotype; OG, other genotype; CGF, coded genotype frequency; β_GE_, effect size of interaction term; SE, standard error.

The sentinel variant rs8022678 was located at a gene desert region between the *BCL11B* and *VRK1* genes (Supplementary Fig. S7). Expression quantitative trait locus (eQTL) analysis using Japanese multi-omics iMETHYL datasets^25,32,33^ showed that the rs8022678 A allele was weakly associated with decreased *BCL11B* expression level in CD4^+^ T cells (Supplementary Fig. S8).

### Relationship between rs8022678 genotype, daily sodium consumption and BP

We stratified our populations according to rs8022678 genotype (GG homo or A carrier). In the discovery cohort, 48.0% of subjects were rs8022678 A carriers. The proportion of rs8022678 A carriers was 45.1% in the replication cohort. In both cohorts, no significant differences in age, BMI, daily sodium consumption, SBP, DBP, proportion of females, proportion of hypertensive subjects, or proportion of antihypertensive medication were observed between rs8022678 A carriers and non-carriers (Supplementary Table S11).

**Figure 1:**
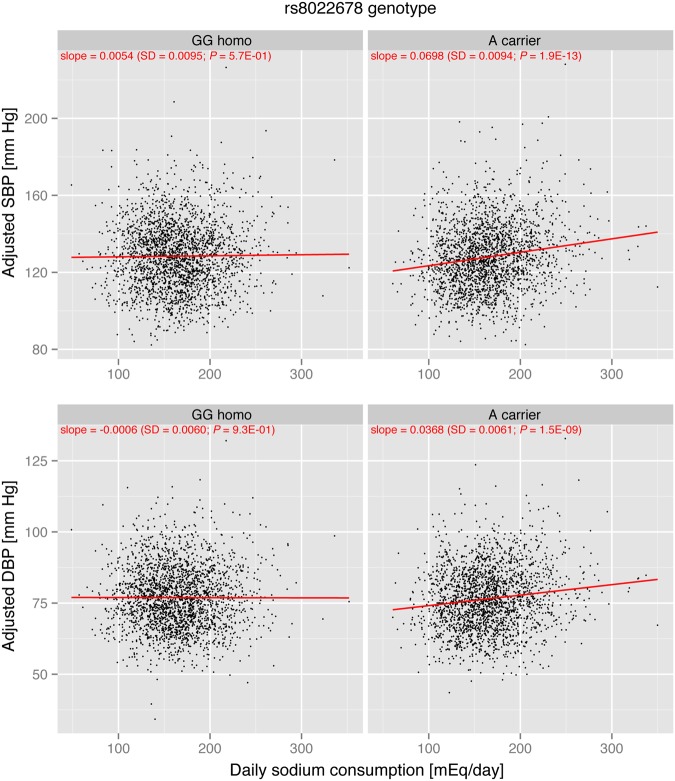
Sodium effect on blood pressure stratified by rs8022678 genotype in the discovery cohort. The *x*-axis indicates daily sodium consumption level. The *y*-axis shows age-, sex-, and BMI-adjusted blood pressure (top panels, systolic blood pressure; bottom panels, diastolic blood pressure). Plots for rs8022678 A non-carriers (denoted as “GG homo”) are shown in the left-side panels and plots for rs8022678 A carriers (denoted as “A carriers”) are shown in the right-side panels. The red lines represent regression lines, and the slope parameters are shown at the top of each panel in red.

In the discovery cohort, sodium intake effect on SBP and DBP was estimated for each subgroup of rs8022678 A carriers and non-carriers. A linear regression analysis adjusted for age, sex, and BMI showed that daily sodium consumption was not significantly associated with SBP (*P* = 0.57) or DBP (*P* = 0.93) in rs8022678 A non-carriers (Fig. 1). In contrast, daily sodium consumption was positively correlated with SBP (*P* = 1.9 × 10^−13^) and DBP (*P* = 1.5 × 10^−9^) in rs8022678 A carriers. We further stratified the discovery populations according to the tertile of daily sodium consumption (low, medium, and high consumption subgroups). For rs8022678 A non-carriers, SBP and DBP were not significantly different between high and low consumption subgroups (Table 3). For rs8022678 A carriers, mean differences between high and low consumption subgroups were 5.9 mm Hg (*P* = 8.8 × 10^−12^) for SBP and 2.9 mm Hg (*P* = 6.7 × 10^−7^) for DBP.

**Table 3:** Sodium effect on blood pressure stratified by rs8022678 genotype in the discovery cohort.

rs8022678	Variable	Tertile of daily sodium consumption		
		T1 (Low)	T2 (Medium)	T3 (High)
All	Range, mEq/day (mg/day)	<147.5 (<3,393)	147.5–178.0 (3,393–4,094)	>178.0 ( > 4,094)
	SBP (mean ± SD), mm Hg	127.2 ± 16.8	127.6 ± 16.9	129.8 ± 17.8
	Mean difference in SBP, mm Hg	ref.	0.4	2.7
	*P*	ref.	5.0 × 10^−1^	1.2 × 10^−4^
	DBP (mean ± SD), mm Hg	76.3 ± 10.7	76.5 ± 10.7	77.5 ± 11.3
	Mean difference in DBP, mm Hg	ref.	0.2	1.2
	*P*	ref.	7.2 × 10^−1^	6.7 × 10^−3^
GG homo	SBP (mean ± SD), mm Hg	129.1 ± 17.0	127.2 ± 17.0	128.8 ± 17.8
	Mean difference in SBP, mm Hg	ref.	−1.9	−0.3
	*P*	ref.	2.0 × 10^−2^	2.7 × 10^−1^
	DBP (mean ± SD), mm Hg	77.4 ± 10.7	76.3 ± 10.7	77.0 ± 11.1
	Mean difference in DBP, mm Hg	ref.	−1.1	−0.4
	*P*	ref.	2.4 × 10^−2^	2.7 × 10^−1^
A carrier	SBP (mean ± SD), mm Hg	125.1 ± 16.3	128.0 ± 16.7	130.9 ± 17.7
	Mean difference in SBP, mm Hg	ref.	3.0	5.9
	*P*	ref.	5.1 × 10^−4^	8.8 × 10^−12^
	DBP (mean ± SD), mm Hg	75.0 ± 10.5	76.6 ± 10.6	77.9 ± 11.5
	Mean difference in DBP, mm Hg	ref.	1.6	2.9
	*P*	ref.	3.8 × 10^−3^	6.7 × 10^−7^

SBP, systolic blood pressure; DBP, diastolic blood pressure; SD, standard deviation; ref., reference.

**Figure 2:**
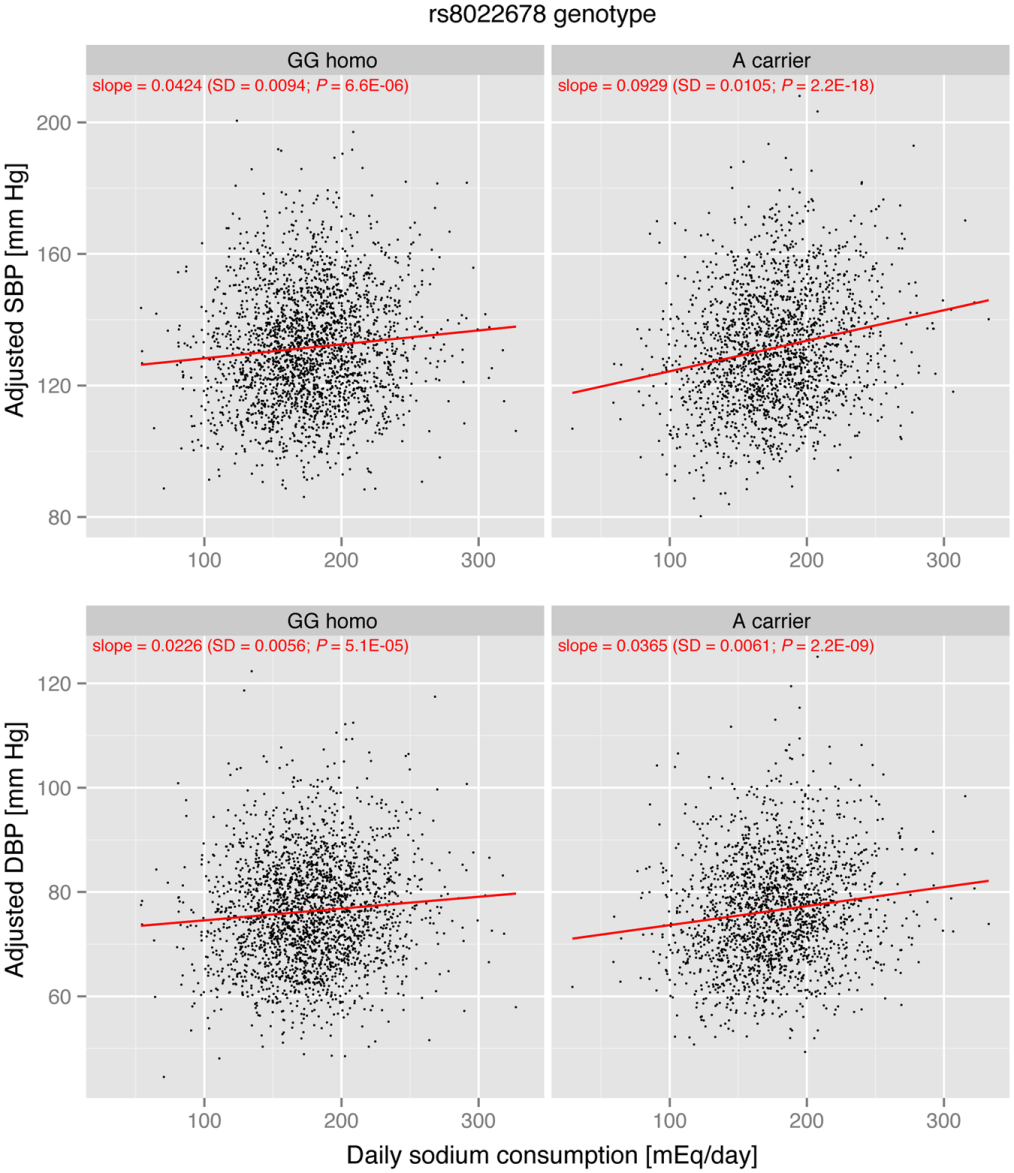
Sodium effect on blood pressure stratified by rs8022678 genotype in the replication cohort. The *x*-axis indicates daily sodium consumption level. The *y*-axis shows age-, sex-, and BMI-adjusted blood pressure (top panels, systolic blood pressure; bottom panels, diastolic blood pressure). Plots for rs8022678 A non-carriers (denoted as “GG homo”) are shown in the left-side panels and plots for rs8022678 A carriers (denoted as “A carriers”) are shown in the right-side panels. The red lines represent regression lines, and the slope parameters are shown at the top of each panel in red.

In the replication cohort, sodium consumption effect on BP was significant in both rs8022678 A carriers and non-carriers. However, effect size estimates in rs8022678 A carriers (β = 0.093 [*P* = 2.2 × 10^−18^] for SBP and β = 0.037 [*P* = 2.2 × 10^−9^] for DBP) were markedly larger than those in rs8022678 A non-carriers (β = 0.042 [*P* = 6.6 × 10^−6^] for SBP and β = 0.023 [*P* = 5.1 × 10^−5^] for DBP) (Fig. 2). Mean differences between high and low consumption subgroups were 6.9 mm Hg SBP (*P* = 2.6 × 10^−10^) and 2.8 mm Hg DBP (*P* = 2.7 × 10^−6^) in rs8022678 A carriers, which were apparently larger than those in rs8022678 A non-carriers (3.5 mm Hg SBP [*P* = 4.9 × 10^−5^] and 1.8 mm Hg DBP [*P* = 7.1 × 10^−4^]; Table 4).

**Table 4:** Sodium effect on blood pressure stratified by rs8022678 genotype in the replication cohort.

rs8022678	Variable	Tertile of daily sodium consumption		
		T1 (Low)	T2 (Medium)	T3 (High)
All	Range, mEq/day (mg/day)	<159.9 (<3,678)	159.9–191.7 (3,678–4,409)	>191.7 (>4,409)
	SBP (mean ± SD), mm Hg	128.9 ± 17.7	131.6 ± 17.6	133.9 ± 17.6
	Mean difference in SBP, mm Hg	ref.	2.7	5.0
	*P*	ref.	7.8 × 10^−5^	3.6 × 10^−13^
	DBP (mean ± SD), mm Hg	75.2 ± 10.3	76.4 ± 10.2	77.5 ± 10.4
	Mean difference in DBP, mm Hg	ref.	1.2	2.3
	*P*	ref.	2.9 × 10^−3^	1.6 × 10^−8^
GG homo	SBP (mean ± SD), mm Hg	129.7 ± 17.7	131.7 ± 17.1	133.2 ± 17.3
	Mean difference in SBP, mm Hg	ref.	2.0	3.5
	*P*	ref.	1.1 × 10^−2^	4.9 × 10^−5^
	DBP (mean ± SD), mm Hg	75.5 ± 10.4	76.1 ± 10.1	77.3 ± 10.4
	Mean difference in DBP, mm Hg	ref.	0.7	1.8
	*P*	ref.	2.1 × 10^−1^	7.1 × 10^−4^
A carrier	SBP (mean ± SD), mm Hg	128.0 ± 17.6	131.5 ± 18.1	134.9 ± 18.1
	Mean difference in SBP, mm Hg	ref.	3.5	6.9
	*P*	ref.	2.1 × 10^−3^	2.6 × 10^−10^
	DBP (mean ± SD), mm Hg	74.9 ± 10.3	76.7 ± 10.2	77.7 ± 10.4
	Mean difference in DBP, mm Hg	ref.	1.8	2.8
	*P*	ref.	2.3 × 10^−3^	2.7 × 10^−6^

SBP, systolic blood pressure; DBP, diastolic blood pressure; SD, standard deviation; ref., reference.

These tendencies were robust against sensitivity analyses. The analysis of measured rather than imputed BP values, exclusion of subjects taking antihypertensive medication, or exclusion of hypertensive subjects did not alter the results (Supplementary Figs S9–S14 and Supplementary Tables S12–S17).

## Discussion

We estimated sodium intake level of the study participants using a spot urine samples and the verified Tanaka’s formula^20^, conducted genome-wide analyses to search for genetic factors that influence the effects of sodium on BP, and identified a novel 3′-*BCL11B* gene desert locus. Our detection criteria were comparable to or slightly more stringent than those of a previous genome-wide interaction study^18^. In addition, sensitivity analyses did not alter the relationship between rs8022678 (the sentinel variant of the 3′*-BCL11B* gene desert locus), daily sodium consumption, and BP. Accordingly, rs8022678 is likely a genuine genetic factor that modulates the effects of sodium on BP.

Previous studies indicated that the 3′-*BCL11B* gene desert region harbours several remote enhancers that modulate *BCL11B* gene expression^34,35^. Japanese multi-omics iMETHYL datasets^25,32,33^ showed that the rs8022678 A allele was weakly associated with decreased *BCL11B* expression level in CD4^+^ T cells. Some variants at the 3′-*BCL11B* gene desert region were associated with aortic stiffness and cardiovascular disease risk^36^, although rs8022678 was not linked with those variants by linkage disequilibrium (*r*^2^ = 0.002 in East Asians according to the LDlink server^37^). *BCL11B* encodes a lineage-specific transcription factor that is important for the differentiation of double-positive thymocytes into CD4 or CD8 single-positive (SP) T cells^38–40^. Moreover, BCL11B is expressed in CD4^+^ SP lymphocytes to control gene expression of interleukin-2 (IL-2), playing a central role in T cell proliferation^41^. Interestingly, emerging experimental evidence shows the involvement of T cells in the pathogenesis of salt-sensitive hypertension^42,43^. In rodents, T cells infiltrate the kidney during experimental hypertension^44^, promoting kidney injury via the generation of reactive oxygen species and proinflammatory cytokines, such as IL-17A, IFN-γ, and TNF-α^43,45^. Such injury can disrupt the nephron’s capacity to properly excrete sodium and water, resulting in elevated BP^43^. In humans, accumulation of immune cells was observed in the kidneys of hypertensive patients^46^. Taken together, our finding that a novel 3′-*BCL11B* gene desert locus affects the effect of sodium on BP may indicate that BCL11B is a key molecule involved in the pathogenesis of salt-sensitive hypertension, possibly via modulating differentiation and proliferation of proinflammatory T cells.

The large number of subjects in our investigation, which was threefold higher than that in a previous genome-wide interaction study^18^, was essential for the detection of the 3′- *BCL11B* gene desert locus. From the analyses of the discovery cohort, effect size estimates of daily sodium consumption (β_E_) and the term of rs8022678 × sodium interaction (β_GE_) for SBP were 0.041 and 0.064, respectively. Therefore, the ratio between those effect size values (β_GE_/β_E_) was 1.56. The power to detect interactions with the effect size ratio of 1.5 and effect genotype frequency of 0.5 was estimated to be 57.5% under the dominant or recessive model, indicating that the large sample size of our cohorts enabled for the first time to discover the rs8022678 × sodium interaction.

In a previous genome-wide interaction study, polymorphism × sodium interaction was tested only under the dosage model^18^. However, candidate gene studies have reported that several genetic variations exerted dominant or recessive effects^15–17^. Accordingly, we additionally tested the interactions under additive, dominant, and recessive models. As a result, the 3′*-BCL11B* gene desert locus was discovered only in the recessive model, indicating the importance to consider dominant and recessive models for genome-wide interaction analyses.

The sodium effect on BP was clearly different between subgroups defined by the rs8022678 genotype. Both in the discovery and replication cohorts, stronger sodium effects on BP were observed in the subgroup of rs8022678 A carriers than in the subgroup of rs8022678 A non-carriers. These results suggest that the rs8022678 genotype can classify individuals into sodium-sensitive (rs8022678 A carriers) and sodium-insensitive (rs8022678 A non-carriers) subgroups. Interestingly, rs8022678 A carriers showed higher BP than rs8022678 A non-carriers in the subgroup of high sodium consumption, whereas rs8022678 A carriers showed lower BP than rs8022678 A non-carriers in the subgroup of low sodium consumption (Tables 3 and 4). In addition, rs8022678 A carriers had a lower probability to present with hypertension than rs8022678 A non-carriers among the subjects who consumed low levels of sodium (odds ratio [OR], 0.80; 95% confidence interval [CI], 0.68–0.94; *P* = 0.006), whereas rs8022678 A carriers had a slightly higher probability to be hypertensive than rs8022687 A non-carriers among the subjects who consumed high levels of sodium (OR, 1.14; 95% CI, 0.98–1.32; *P* = 0.10) (Supplementary Table S18). These data indicate that rs8022678 A carriers, compared to rs8022678 A non-carriers, are exposed to a higher risk of elevated BP if they consume large amounts of sodium, but they can receive a greater benefit from a lifestyle that consumes low amounts of sodium.

We estimated that the mean SBP difference between high and low sodium consumption subgroups was 5.9 to 6.9 mm Hg in rs8022678 A carriers, which was remarkably larger than that in rs8022678 A non-carriers (−0.3 to 3.5 mm Hg) and in the overall population (2.3 to 2.7 mm Hg). Such a large SBP difference in rs8022678 A carriers leads to a hypothesis that interventions to lower sodium consumption might achieve greater reduction of BP in rs8022678 A carriers than in rs8022678 A non-carriers. To test this hypothesis, future intervention studies are warranted. If the hypothesis will be proven, as rs8022678 A carriers are common in population (45–48% in our cohorts), high-risk approaches that target rs8022678 A carriers would potentially impact the reduction of population prevalence rates of hypertension and cardiovascular disease.

In addition, a recent randomised controlled trial reported that the disclosure of genetic information regarding sodium vulnerability is arguably useful to facilitate changes in an individual’s habits and to promote a reduction in sodium consumption levels^47^. Therefore, high-risk approaches using genotype information may be effective in two ways: (i) identifying persons that can receive greater benefits from reducing sodium consumption, and (ii) improving the degree of sodium reduction achieved by interventions by additionally motivating lifestyle changes through genetic counselling.

A limitation of this study was that we estimated the daily sodium consumption level from a casual spot urine specimen using the Tanaka’s formula^20^. Although the equation had been validated and the 24-h urinary excretion level estimated by the formula correlated well with a gold standard level measured from the 24-h urinary collection, this approximate estimation of daily sodium consumption levels may attenuate the effect size and significance of the interaction term in statistical analyses. It should be noted that effect size values estimated in this study might be underestimated. In addition, large parts of inter-individual variability of BP remained to be explained even after having considered age, sex, BMI, daily sodium consumption, rs8022678 and rs8022678 polymorphism × sodium interaction as explanatory variables (Figs 1 and 2). Potential sources of the inter-individual variability might include genetic factors that influence BP, because BP traits have a substantial genetic component (heritability estimates range from 31% to 68%)^48^. Another limitation was that our sample size could not allow the detection of relatively weak interactions, for which the effect size ratio (β_GE_/β_E_) is less than 1. Future large-scale genome-wide interaction analyses and meta-analyses are needed to identify interactions with small effect sizes.

In summary, through the largest-scale genome-wide analysis to date, we identified a novel 3′-*BCL11B* gene desert polymorphism × sodium interaction that influences BP. Our data showed that rs8022678 genotype can classify individuals into sodium-sensitive (A carriers) and sodium-insensitive (A non-carriers) subgroups. This finding suggests that rs8022678 A carriers, compared to non-carriers, may receive a greater benefit from sodium-lowering interventions. Our results implied a possibility that high-risk group targeting approaches using the rs8022678 genotype may have an impact on reducing the prevalence of hypertension and cardiovascular disease.

## Electronic supplementary material

Supplementary information

## Data Availability

The datasets analysed in the current study are not publicly available for ethical reasons but are available upon request after approval from the Ethical Committee of Iwate Medical University, the Ethical Committee of Tohoku University, and the Materials and Information Distribution Review Committee of the TMM Project.
