# Supplementary material for: Genome-wide analysis of polymorphism × sodium interaction effect on blood pressure identifies a novel 3′-BCL11B gene desert locus
Source: Sci Rep. 2018 Sep 21;8:14162. doi: 10.1038/s41598-018-32074-1 (PMC6155053; doi:10.1038/s41598-018-32074-1)
Supplement: Supplementary file 1 — Supplementary information [file 41598_2018_32074_MOESM1_ESM.pdf]

Supplementary information for 'Genome-wide analysis of polymorphism × sodium interaction effect on blood pressure identifies a novel 3′-*BCL11B* gene desert locus'

Tsuyoshi Hachiya, Akira Narita, Hideki Ohmomo, Yoichi Sutoh, Shohei Komaki, Kozo Tanno, Mamoru Satoh, Kiyomi Sakata, Jiro Hitomi, Motoyuki Nakamura, Kuniaki Ogasawara, Masayuki Yamamoto, Makoto Sasaki, Atsushi Hozawa and Atsushi Shimizu

Supplementary Figures S1 to S14
Supplementary Tables S1 to S18

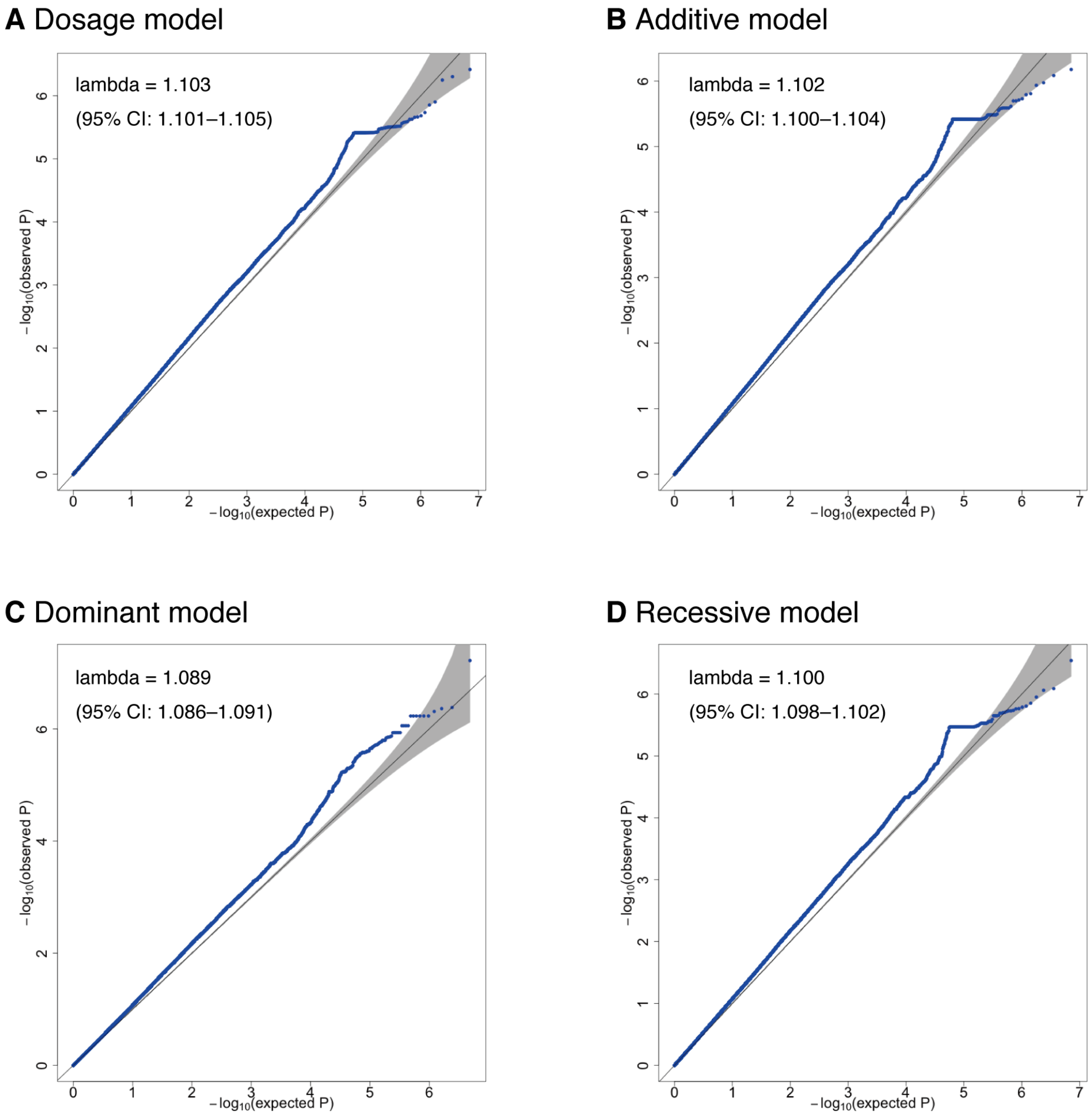

Figure S1. Quantile-quantile plots of genome-wide interaction analyses for SBP before applying genomic control correction.

The *x*-axis indicates the expected $-\log_{10}$ *P*-values under the null hypothesis. The *y*-axis shows the observed $-\log_{10}$ *P*-values calculated from genome-wide interaction analyses. The black line represents $y = x$, which corresponds to the null hypothesis. The gray shaded area shows 95% confidence interval of the null hypothesis. The genomic inflation factor (lambda) is the median of the observed test statistics divided by the median of the expected test statistics.

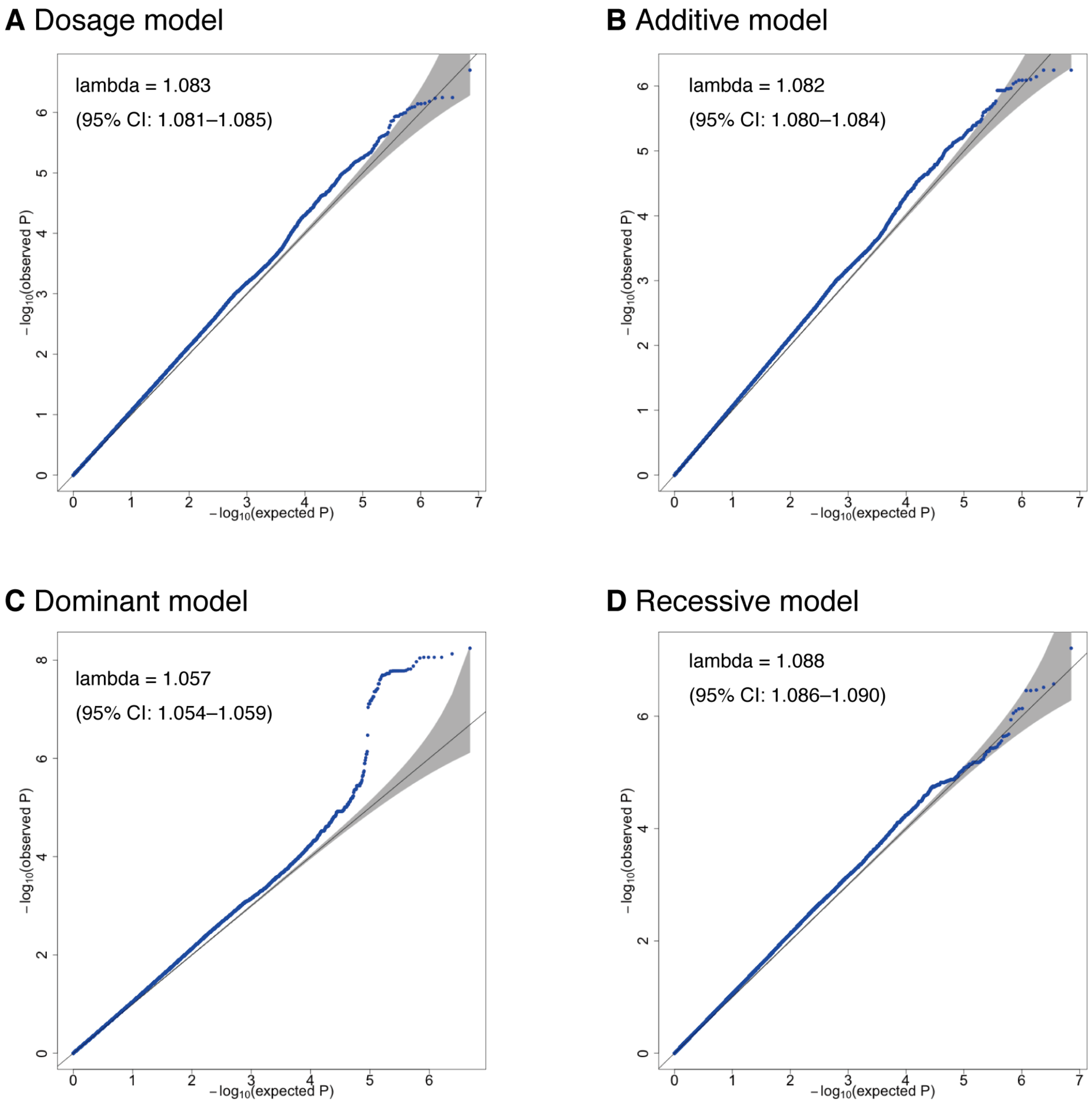

Figure S2. Quantile-quantile plots of genome-wide interaction analyses for DBP before applying genomic control correction.

The *x*-axis indicates the expected $-\log_{10}$ *P*-values under the null hypothesis. The *y*-axis shows the observed $-\log_{10}$ *P*-values calculated from genome-wide interaction analyses. The black line represents $y = x$, which corresponds to the null hypothesis. The gray shaded area shows 95% confidence interval of the null hypothesis. The genomic inflation factor (lambda) is the median of the observed test statistics divided by the median of the expected test statistics.

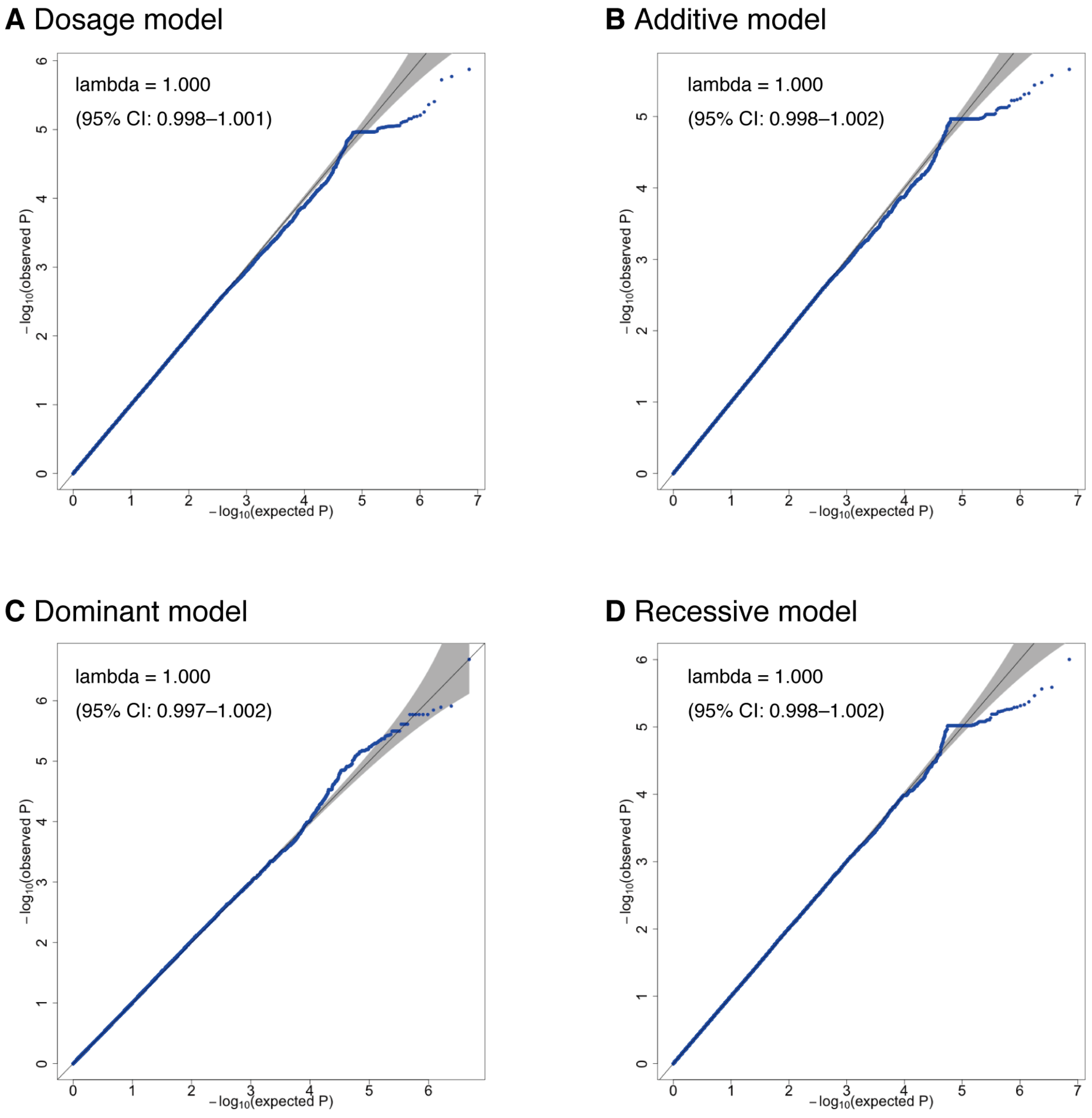

Figure S3. Quantile-quantile plots of genome-wide interaction analyses for SBP after applying genomic control correction.

The *x*-axis indicates the expected $-\log_{10}$ *P*-values under the null hypothesis. The *y*-axis shows the observed $-\log_{10}$ *P*-values calculated from genome-wide interaction analyses. The black line represents $y = x$, which corresponds to the null hypothesis. The gray shaded area shows 95% confidence interval of the null hypothesis. The genomic inflation factor (lambda) is the median of the observed test statistics divided by the median of the expected test statistics.

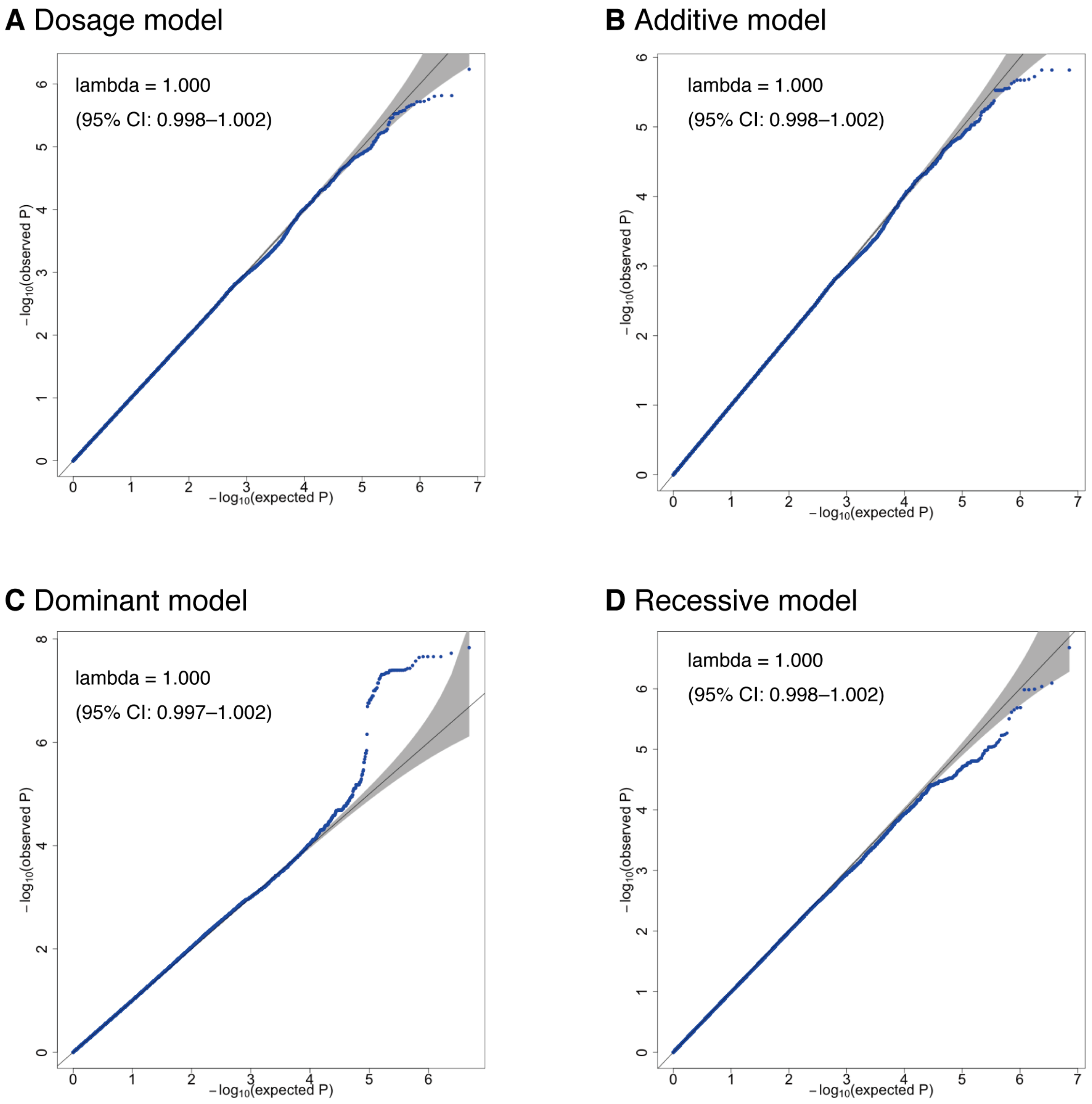

Figure S4. Quantile-quantile plots of genome-wide interaction analyses for DBP after applying genomic control correction.

The *x*-axis indicates the expected $-\log_{10}$ *P*-values under the null hypothesis. The *y*-axis shows the observed $-\log_{10}$ *P*-values calculated from genome-wide interaction analyses. The black line represents $y = x$, which corresponds to the null hypothesis. The gray shaded area shows 95% confidence interval of the null hypothesis. The genomic inflation factor (lambda) is the median of the observed test statistics divided by the median of the expected test statistics.

A Dosage model

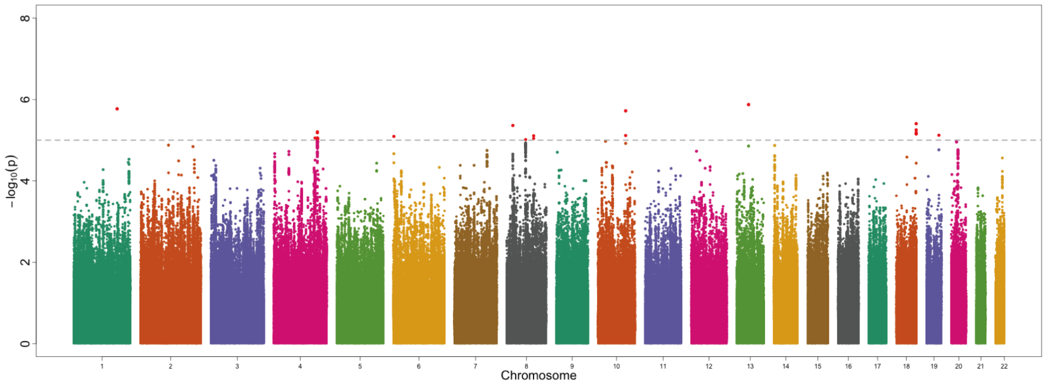

B Additive model

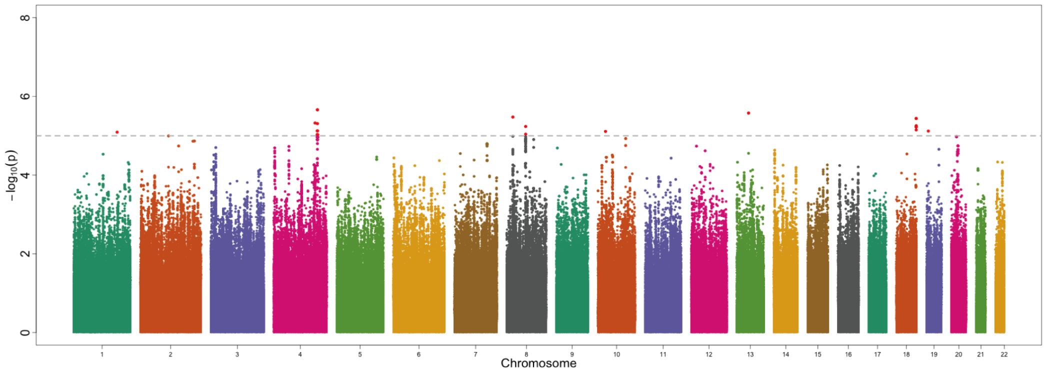

C Dominant model

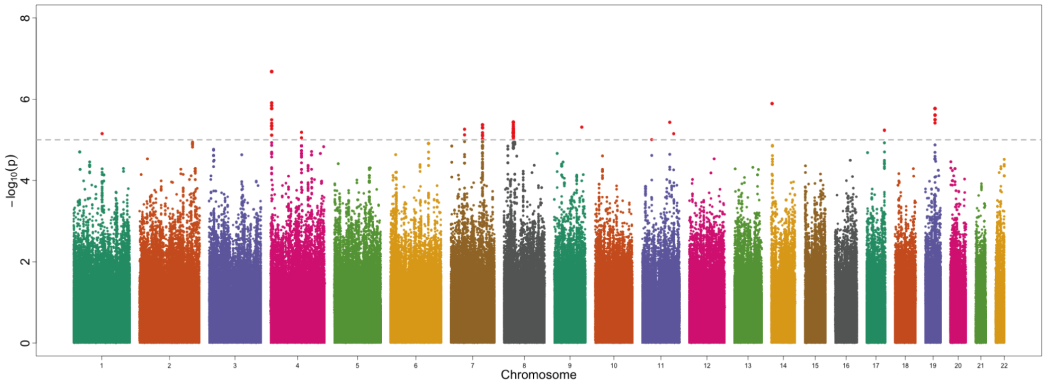

D Recessive model

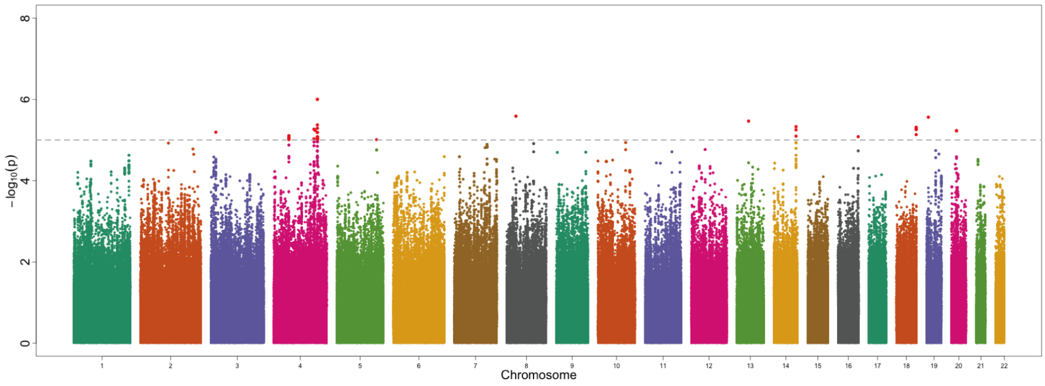

Figure S5. Genome-wide interaction signals for SBP. The *x*-axis represents chromosomal positions and the *y*-axis represents $-\log_{10}$ *P*-values of interaction test. The *P*-values after applying genomic control correction was shown. The grey dotted horizontal lines indicate the suggestive significance level ($P = 1 \times 10^{-5}$). Variants, whose *P*-value was lower than the suggestive significance, were shown in red, whereas colors for other variants indicate chromosomes.

A Dosage model

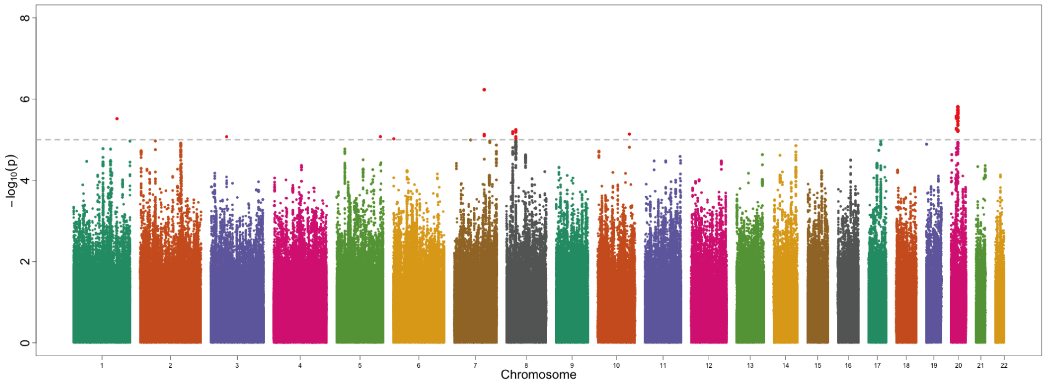

B Additive model

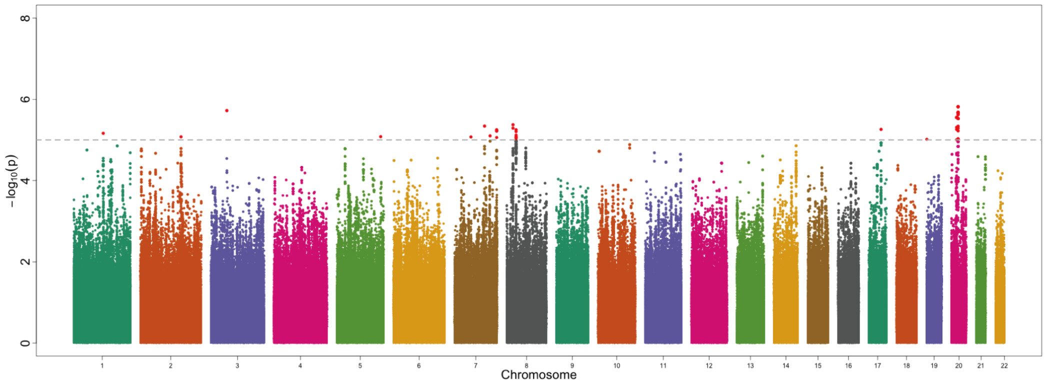

C Dominant model

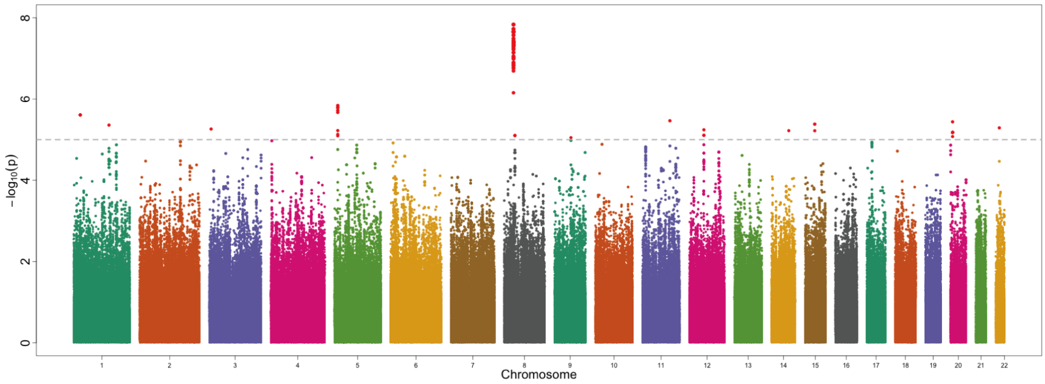

D Recessive model

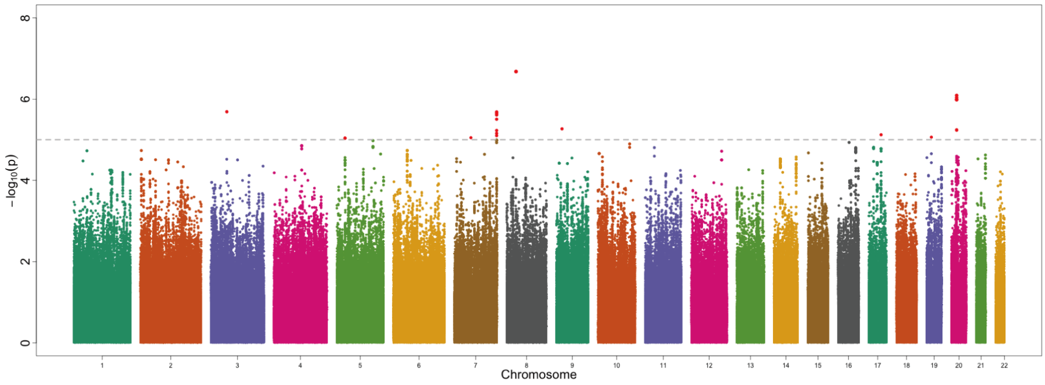

Figure S6. Genome-wide interaction signals for DBP. The *x*-axis represents chromosomal positions and the *y*-axis represents $-\log_{10}$ *P*-values of interaction test. The *P*-values after applying genomic control correction was shown. The grey dotted horizontal lines indicate the suggestive significance level ($P = 1 \times 10^{-5}$). Variants, whose *P*-value was lower than the suggestive significance, were shown in red, whereas colors for other variants indicate chromosomes.

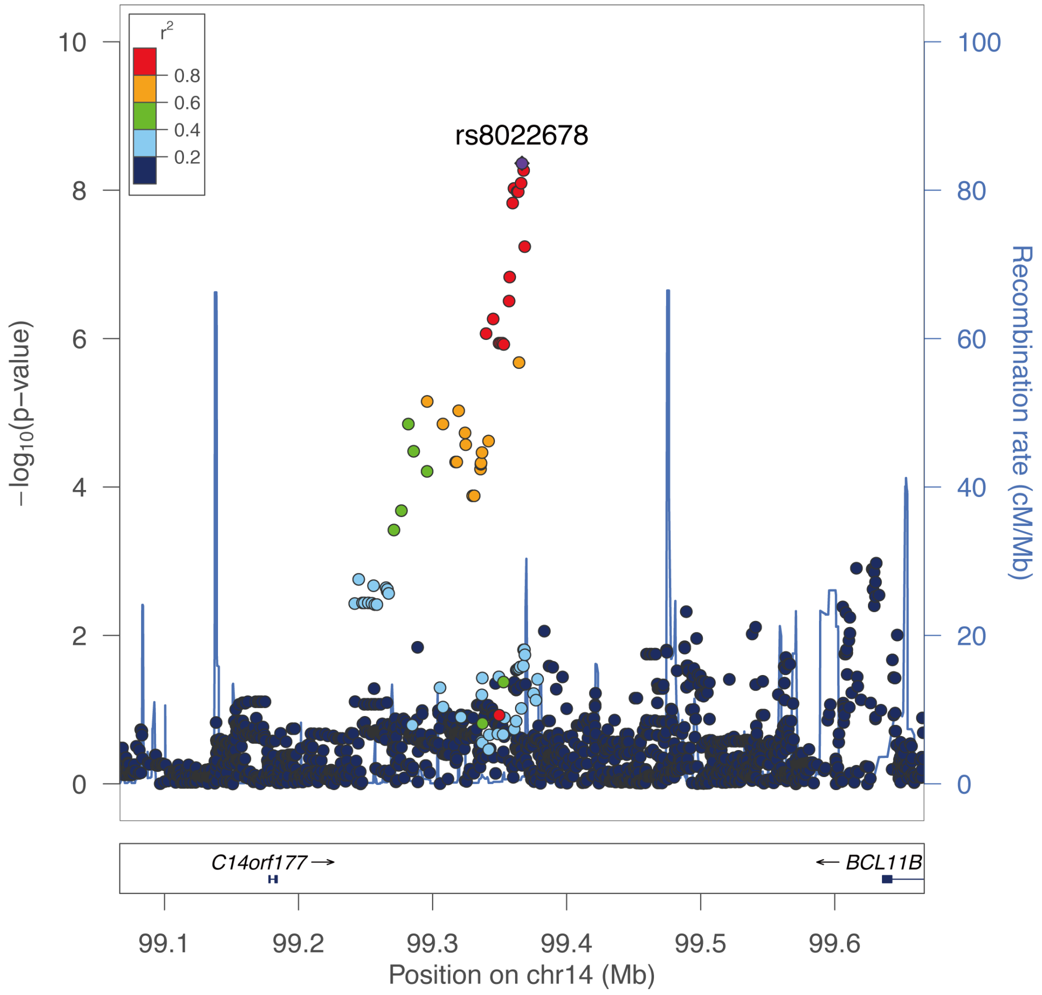

Figure S7. Interaction signals around the 3'-*BCL11B* gene desert locus.

The *x*-axis represents chromosomal positions and the *y*-axis represents $-\log_{10}$ *P*-values. *P*-values in this figure were calculated based on the combined discovery and replication datasets. The lead variant, rs8022678, is shown in purple. Colors represent the degree of linkage disequilibrium (r^2) between each variant and rs8022678.

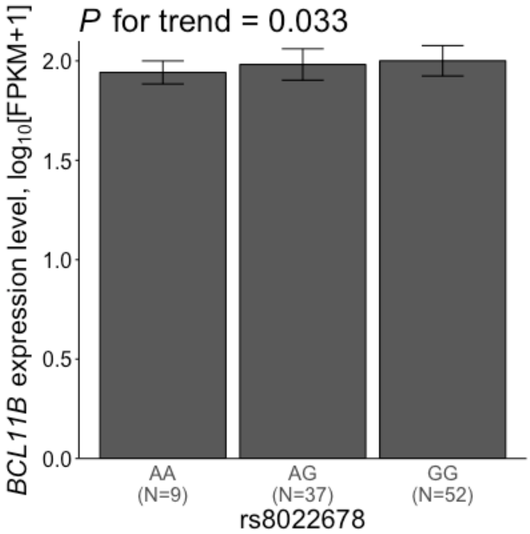

Figure S8. eQTL association between rs8022678 and *BCL11B* in CD4$^+$ T cells. The *x*-axis represents rs8022678 genotype and the *y*-axis represents *BCL11B* gene expression level (in terms of log_{10}[FPKM+1]) in CD4$^+$ T cells. *P*-value for this eQTL association is shown at the top of figure. Bar height shows the mean expression level and error-bars represent standard deviation.

eQTL, expression quantitative trait locus; FPKM, fragments per kilobase of exon per million mapped fragments

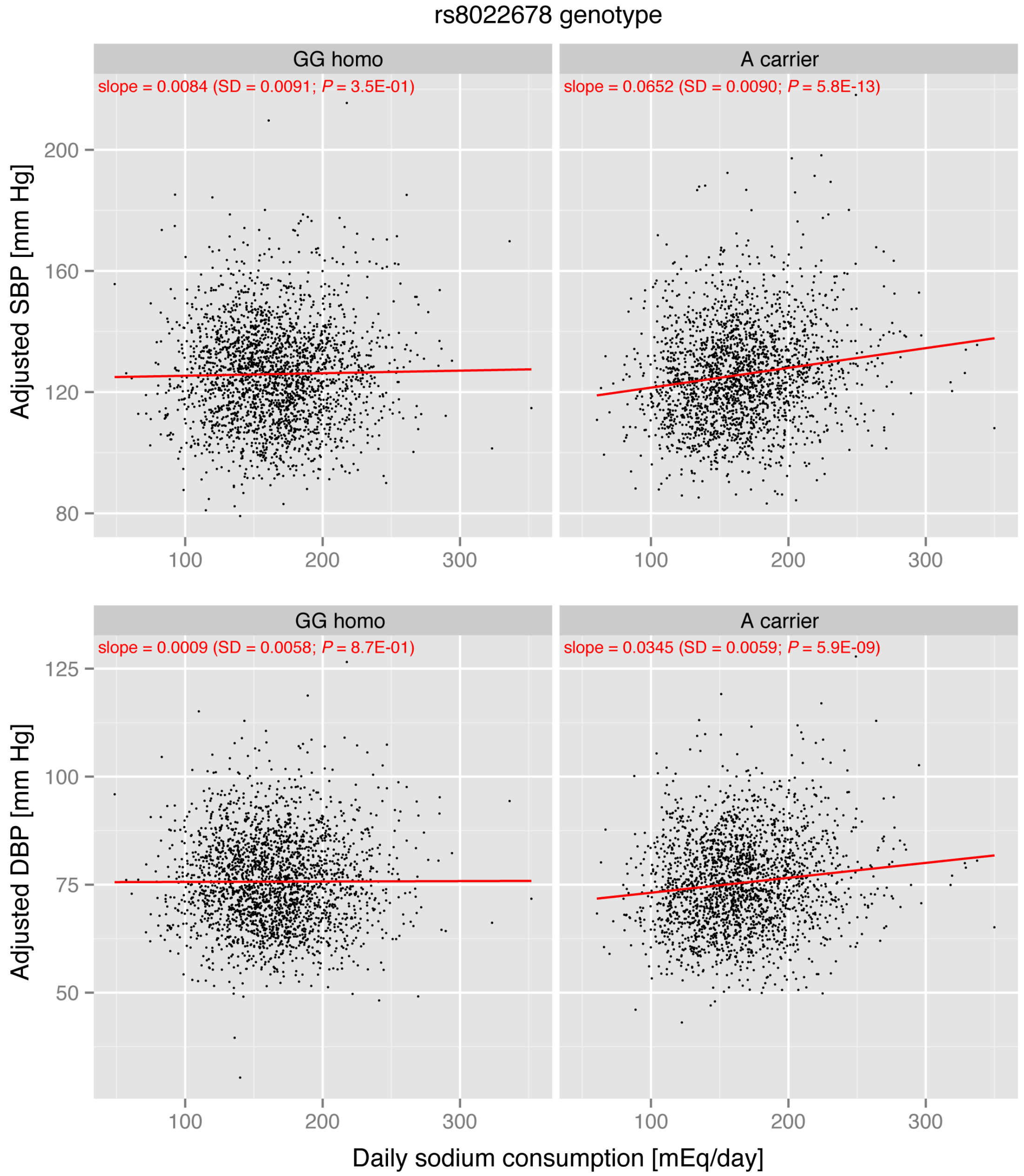

Figure S9. Sodium effect on blood pressure stratified by rs8022678 genotype in discovery cohort. The *x*-axis indicates daily sodium consumption level. The *y*-axis shows age, sex and BMI-adjusted blood pressure (SBP for top panels and DBP for bottom panels). We analyzed measured SBP and DBP rather than imputed SBP and DBP. Plots for rs8022678 A non-carriers (denoted as ‘GG homo’) are shown in left-side panels and plots for rs8022678 A carriers (denoted as ‘A carriers’) are shown in right-side panels. The red lines represent regression lines and the slope parameters are shown at the top of each panel in red.

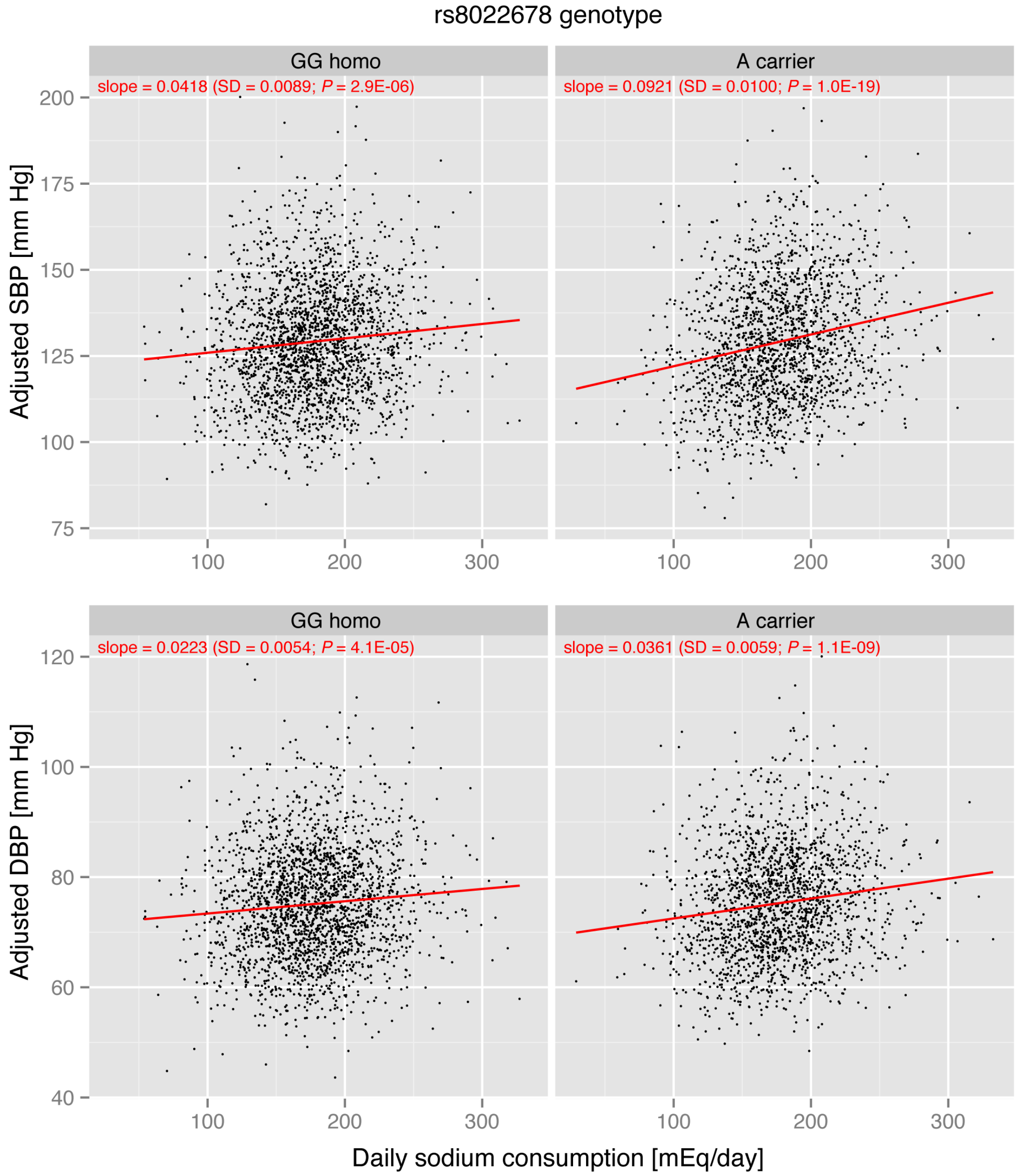

Figure S10. Sodium effect on blood pressure stratified by rs8022678 genotype in replication cohort. The *x*-axis indicates daily sodium consumption level. The *y*-axis shows age, sex and BMI-adjusted blood pressure (SBP for top panels and DBP for bottom panels). We analyzed measured SBP and DBP rather than imputed SBP and DBP. Plots for rs8022678 A non-carriers (denoted as 'GG homo') are shown in left-side panels and plots for rs8022678 A carriers (denoted as 'A carriers') are shown in right-side panels. The red lines represent regression lines and the slope parameters are shown at the top of each panel in red.

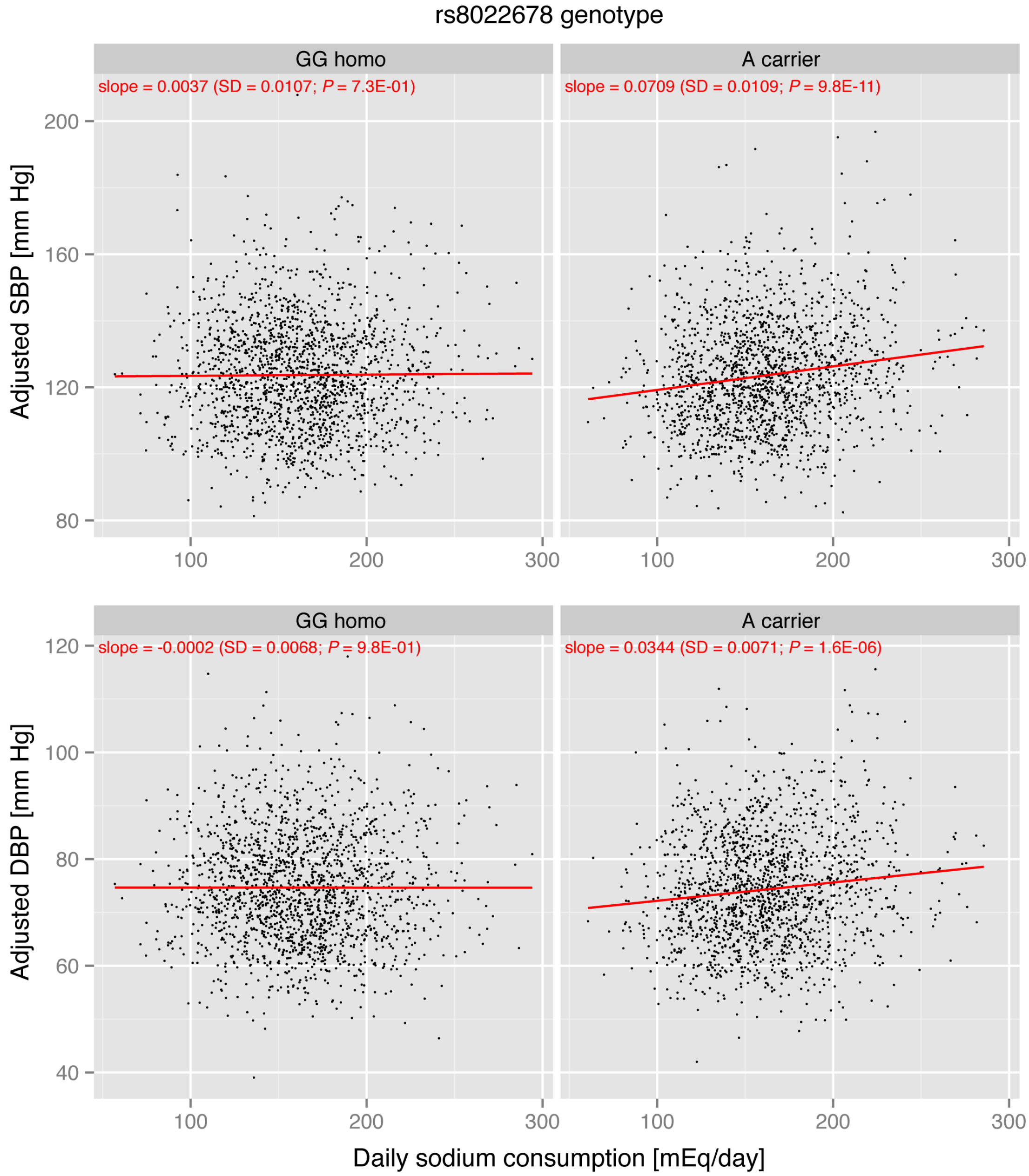

Figure S11. Sodium effect on blood pressure stratified by rs8022678 genotype in discovery cohort with excluding subjects taking antihypertensive medication. The *x*-axis indicates daily sodium consumption level. The *y*-axis shows age, sex and BMI-adjusted blood pressure (SBP for top panels and DBP for bottom panels). Plots for rs8022678 A non-carriers (denoted as 'GG homo') are shown in left-side panels and plots for rs8022678 A carriers (denoted as 'A carriers') are shown in right-side panels. The red lines represent regression lines and the slope parameters are shown at the top of each panel in red.

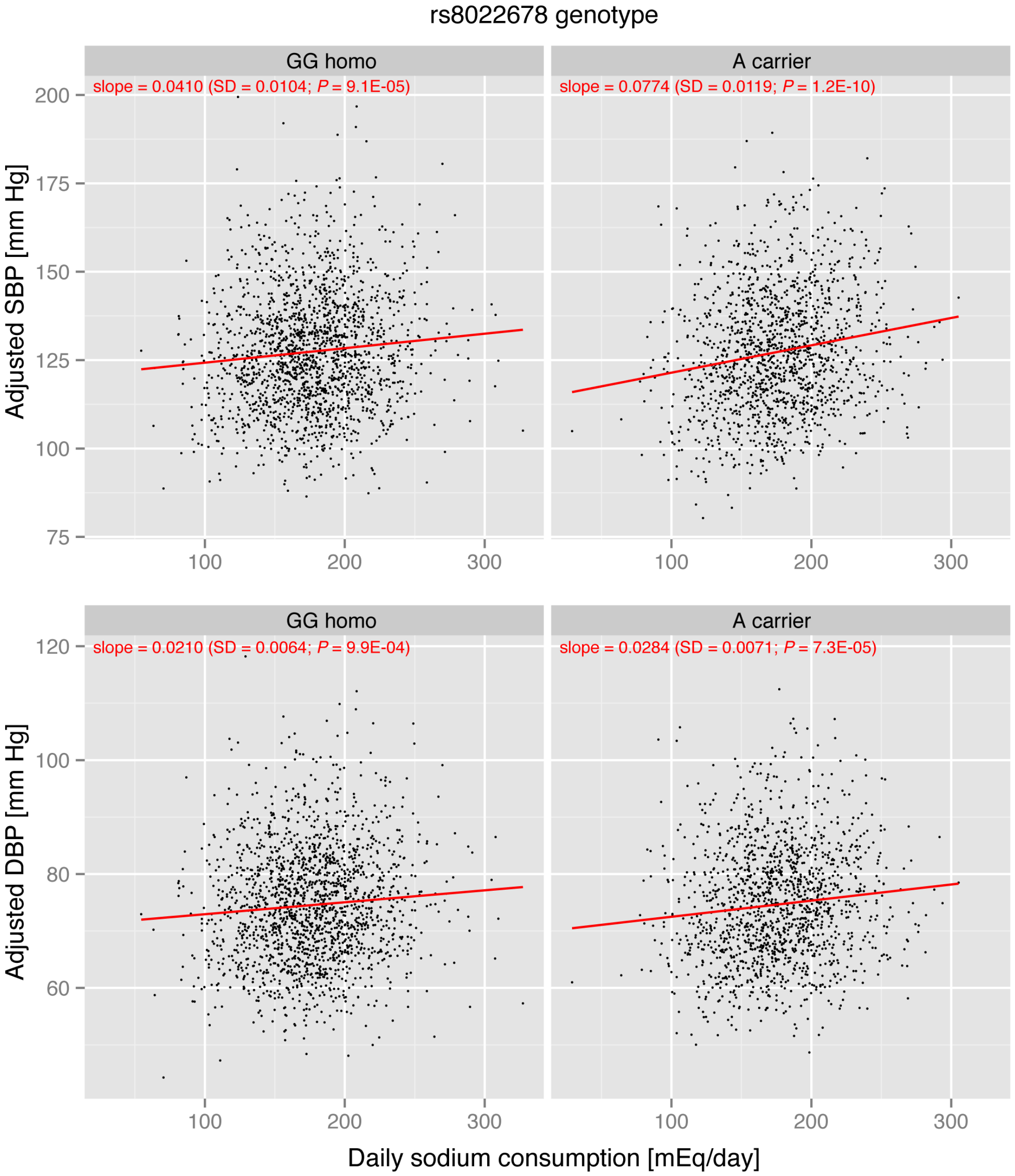

Figure S12. Sodium effect on blood pressure stratified by rs8022678 genotype in replication cohort with excluding subjects taking antihypertensive medication. The *x*-axis indicates daily sodium consumption level. The *y*-axis shows age, sex and BMI-adjusted blood pressure (SBP for top panels and DBP for bottom panels). Plots for rs8022678 A non-carriers (denoted as 'GG homo') are shown in left-side panels and plots for rs8022678 A carriers (denoted as 'A carriers') are shown in right-side panels. The red lines represent regression lines and the slope parameters are shown at the top of each panel in red.

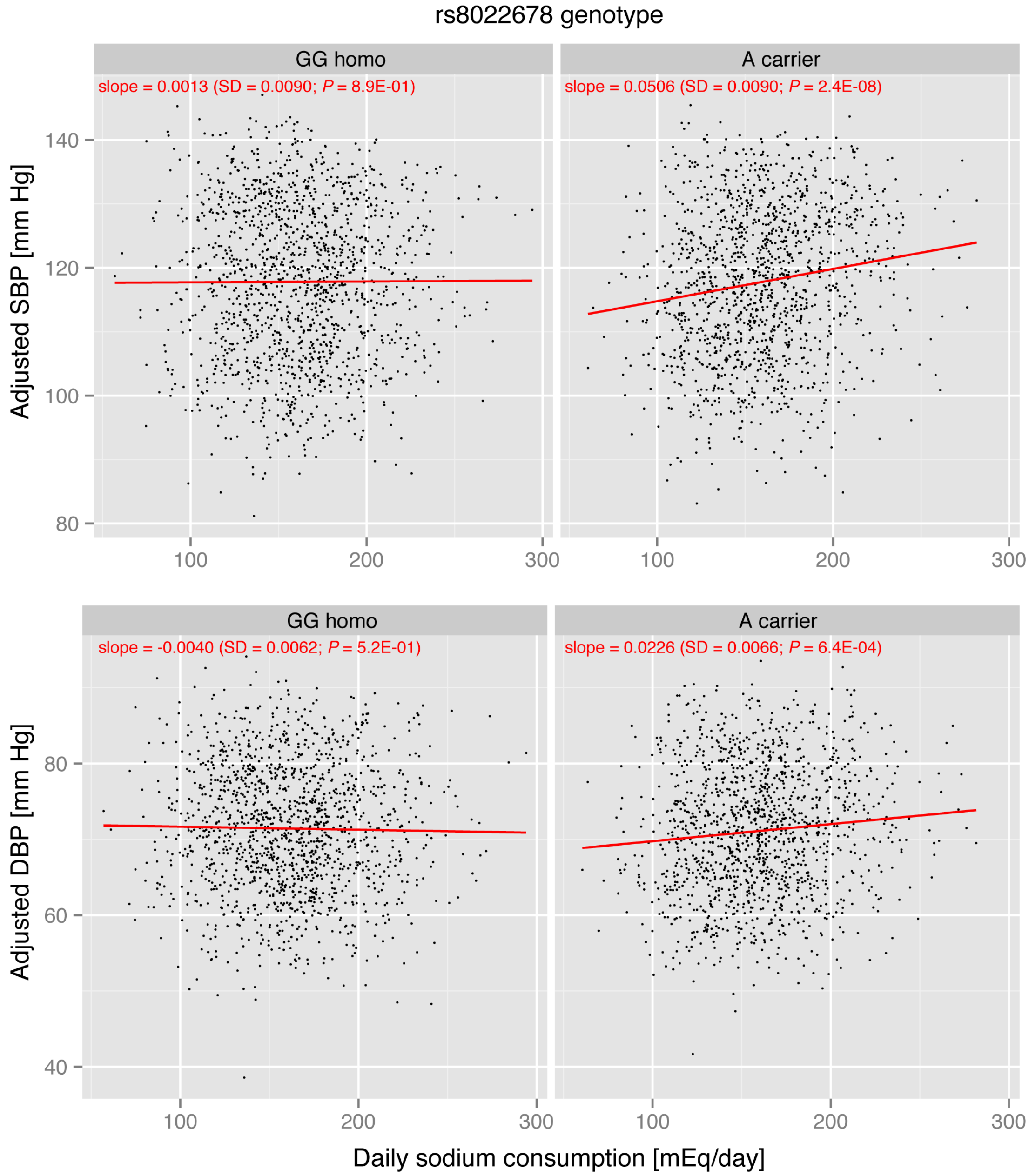

Figure S13. Sodium effect on blood pressure stratified by rs8022678 genotype in discovery cohort with excluding hypertensive subjects. The *x*-axis indicates daily sodium consumption level. The *y*-axis shows age, sex and BMI-adjusted blood pressure (SBP for top panels and DBP for bottom panels). Plots for rs8022678 A non-carriers (denoted as 'GG homo') are shown in left-side panels and plots for rs8022678 A carriers (denoted as 'A carriers') are shown in right-side panels. The red lines represent regression lines and the slope parameters are shown at the top of each panel in red.

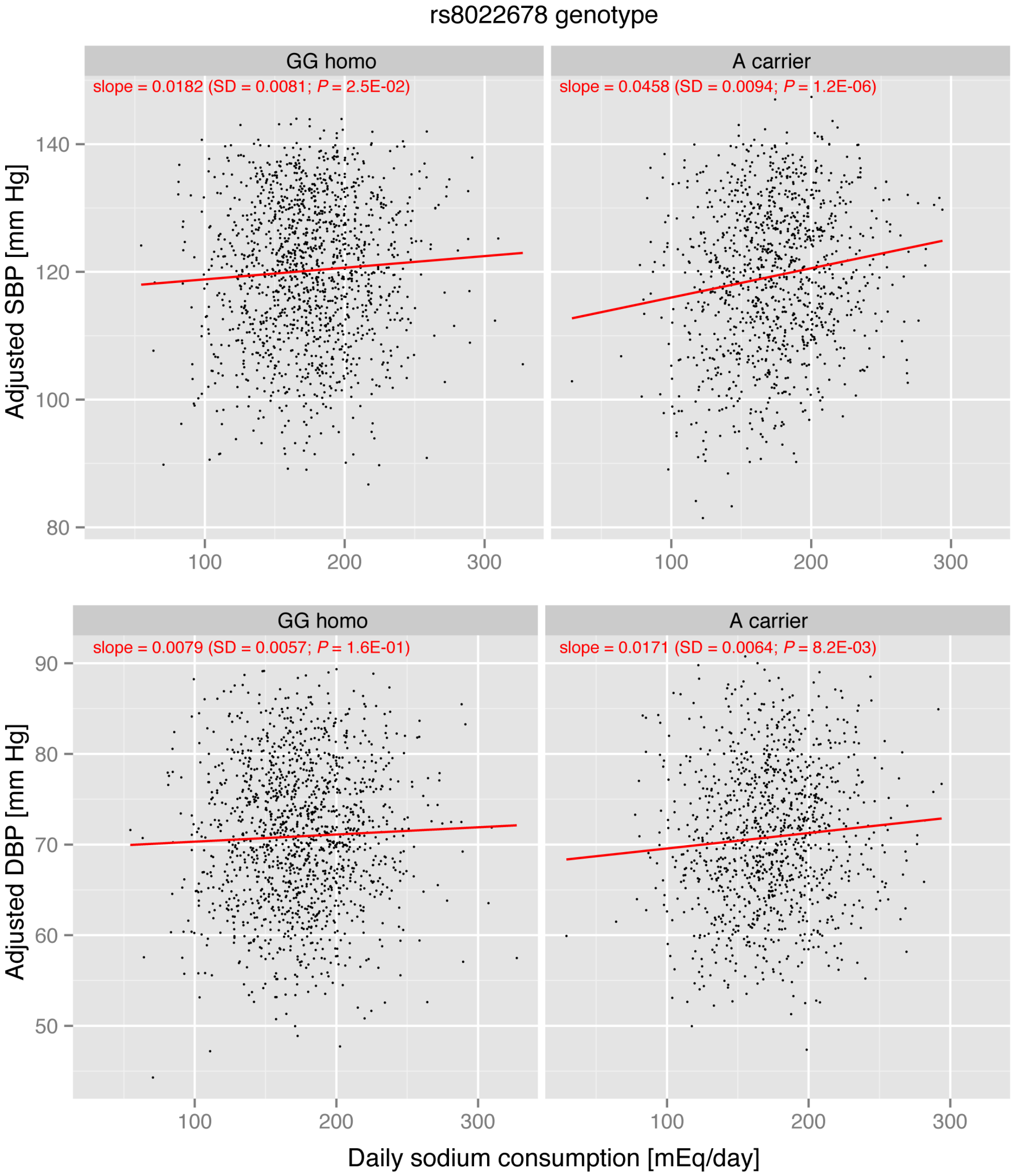

Figure S14. Sodium effect on blood pressure stratified by rs8022678 genotype in replication cohort with excluding hypertensive subjects. The *x*-axis indicates daily sodium consumption level. The *y*-axis shows age, sex and BMI-adjusted blood pressure (SBP for top panels and DBP for bottom panels). Plots for rs8022678 A non-carriers (denoted as 'GG homo') are shown in left-side panels and plots for rs8022678 A carriers (denoted as 'A carriers') are shown in right-side panels. The red lines represent regression lines and the slope parameters are shown at the top of each panel in red.

Table S1. Power calculation for genome-wide interaction analyses

Target variable	SD of target variable*	Sodium effect on target variable†	Model	CAF/ CGF	Effect‡			
					0.5	1.0	1.5	2.0
SBP	17.13512	0.041426	Additive	0.2	0.2	16.2	77.3	99.5
				0.5	1.3	47.5	98.5	100.0
			Dominant/ Recessive	0.2	0.0	2.8	26.1	67.5
				0.5	0.1	9.7	57.5	95.7
DBP	10.88337	0.019897	Additive	0.2	0.2	4.4	30.1	81.0
				0.5	0.0	14.2	69.9	98.2
			Dominant/ Recessive	0.2	0.2	0.4	4.9	23.5
				0.5	0.0	1.9	17.7	56.7

Power was shown in percentage (%). To calculate power, we repeated 1,000 simulations for each parameter set (Alpha = 1E-05; *N* = 4,527).

SBP, systolic blood pressure; DBP, diastolic blood pressure; SD, standard deviation; CAF, coded allele frequency (for additive model); CGF, coded genotype frequency (for dominant/recessive models)

* SD of age, sex and BMI-adjusted residuals of target variables, which was estimated from the discovery cohort

† Difference of age, sex and BMI-adjusted target variables per 1 mEq/day daily sodium consumption, which was estimated from the discovery cohort (β_E)

‡ Ratio of the main effect of sodium and the interaction term (β_{GE}/β_E)

Table S2. Genomic inflation factors with varying the number of principal components

Target variable	Model	#. Principal components				
		0	5	10	15	20
SBP	Dosage	1.109<br>(1.107 – 1.111)	1.111<br>(1.109 – 1.112)	1.105<br>(1.103 – 1.107)	1.103<br>(1.101 – 1.105)	1.103<br>(1.101 – 1.105)
	Additive	1.109<br>(1.107 – 1.111)	1.111<br>(1.108 – 1.112)	1.105<br>(1.103 – 1.107)	1.102<br>(1.100 – 1.104)	1.102<br>(1.100 – 1.104)
	Dominant	1.094<br>(1.092 – 1.097)	1.097<br>(1.095 – 1.099)	1.090<br>(1.088 – 1.092)	1.087<br>(1.085 – 1.089)	1.089<br>(1.086 – 1.091)
	Recessive	1.108<br>(1.106 – 1.110)	1.109<br>(1.107 – 1.111)	1.103<br>(1.101 – 1.104)	1.101<br>(1.099 – 1.103)	1.100<br>(1.098 – 1.102)
DBP	Dosage	1.083<br>(1.081 – 1.085)	1.085<br>(1.084 – 1.087)	1.083<br>(1.081 – 1.084)	1.081<br>(1.079 – 1.082)	1.083<br>(1.081 – 1.085)
	Additive	1.082<br>(1.081 – 1.084)	1.085<br>(1.083 – 1.087)	1.081<br>(1.079 – 1.083)	1.080<br>(1.078 – 1.082)	1.082<br>(1.080 – 1.084)
	Dominant	1.057<br>(1.055 – 1.059)	1.058<br>(1.056 – 1.060)	1.058<br>(1.055 – 1.060)	1.055<br>(1.053 – 1.057)	1.057<br>(1.054 – 1.059)
	Recessive	1.087<br>(1.085 – 1.089)	1.088<br>(1.086 – 1.090)	1.085<br>(1.083 – 1.087)	1.087<br>(1.085 – 1.089)	1.088<br>(1.086 – 1.090)

Genomic inflation factors and their 95% confidence intervals are shown

Table S3: Polymorphisms × sodium interactions influencing SBP detected from genome-wide analysis under dosage model

Variant	Chr	Position	CA	OA	Discovery				Replication				Combined			
					CAF	β_{GE}	SE(β_{GE})	*P*	CAF	β_{GE}	SE(β_{GE})	*P*	CAF	β_{GE}	SE(β_{GE})	*P*
rs145889214	1	201,816,692	A	G	0.987	-0.212	0.042	1.7E-06	0.980	-0.004	0.038	9.1E-01	0.984	-0.087	0.028	1.7E-03
rs79699215	4	152,352,727	T	C	0.986	0.186	0.040	8.8E-06	0.989	-0.004	0.050	9.3E-01	0.987	0.108	0.031	5.4E-04
rs150495698	4	161,349,746	GT	G	0.948	-0.101	0.021	6.2E-06	0.953	0.002	0.023	9.3E-01	0.951	-0.049	0.015	1.7E-03
rs111573571	6	2,811,803	A	G	0.756	-0.056	0.012	8.0E-06	0.770	-0.015	0.012	2.1E-01	0.763	-0.034	0.009	7.9E-05
rs142373259	8	13,843,028	G	C	0.851	-0.064	0.013	4.3E-06	0.847	-0.014	0.014	3.3E-01	0.849	-0.038	0.010	6.2E-05
rs1483178	8	63,129,740	C	T	0.730	-0.049	0.011	9.6E-06	0.732	-0.013	0.011	2.4E-01	0.731	-0.030	0.008	6.5E-05
rs145994209	8	96,671,420	A	T	0.981	-0.170	0.036	7.8E-06	0.985	-0.004	0.044	9.3E-01	0.983	-0.094	0.028	7.2E-04
10:100305536C>T	10	100,305,536	C	T	0.981	0.204	0.041	1.9E-06	0.981	-0.034	0.042	4.2E-01	0.981	0.085	0.029	2.8E-03
rs182765532	13	62,124,175	T	C	0.968	-0.142	0.028	1.3E-06	0.962	-0.031	0.027	2.3E-01	0.965	-0.077	0.019	5.4E-05
rs948955	18	76,063,947	T	C	0.803	-0.059	0.012	3.9E-06	0.786	0.004	0.012	7.7E-01	0.795	-0.028	0.009	1.4E-03
rs143231822	19	49,822,425	G	T	0.955	0.113	0.024	7.5E-06	0.947	-0.001	0.024	9.6E-01	0.951	0.052	0.017	1.8E-03

Chr, chromosome; CA, coded allele; OA, other allele; CAF, coded allele frequency

Table S4: Polymorphisms × sodium interactions influencing SBP detected from genome-wide analysis under additive model

Variant	Chr	Position	CA	OA	Discovery				Replication				Combined			
					CAF	β_{GE}	SE(β_{GE})	*P*	CAF	β_{GE}	SE(β_{GE})	*P*	CAF	β_{GE}	SE(β_{GE})	*P*
rs145889214	1	201,816,692	A	G	0.988	-0.189	0.040	8.1E-06	0.979	-0.008	0.037	8.4E-01	0.983	-0.082	0.027	2.3E-03
rs79699215	4	152,352,727	T	C	0.986	0.177	0.037	4.7E-06	0.988	-0.011	0.047	8.2E-01	0.987	0.103	0.029	4.0E-04
rs199624187	4	161,353,109	AT	A	0.932	-0.090	0.018	2.2E-06	0.932	-0.001	0.019	9.6E-01	0.932	-0.045	0.013	6.4E-04
rs142373259	8	13,843,028	G	C	0.849	-0.063	0.013	3.3E-06	0.845	-0.014	0.014	3.0E-01	0.847	-0.038	0.009	4.2E-05
rs1483178	8	63,129,740	C	T	0.731	-0.050	0.011	5.8E-06	0.733	-0.012	0.011	2.9E-01	0.732	-0.030	0.008	6.5E-05
rs10828408	10	23,372,303	A	G	0.509	-0.044	0.009	7.7E-06	0.480	0.002	0.010	8.0E-01	0.495	-0.023	0.007	7.1E-04
rs182765532	13	62,124,175	T	C	0.968	-0.133	0.027	2.6E-06	0.964	-0.021	0.026	4.0E-01	0.966	-0.067	0.018	2.7E-04
rs948955	18	76,063,947	T	C	0.802	-0.057	0.012	3.6E-06	0.787	0.001	0.012	9.2E-01	0.795	-0.028	0.008	7.9E-04
19:8137604G>GCACACACA	19	8,137,604	G	GCACACACA	0.857	-0.065	0.014	7.6E-06	0.854	0.014	0.014	3.3E-01	0.856	-0.024	0.010	1.3E-02

Chr, chromosome; CA, coded allele; OA, other allele; CAF, coded allele frequency

Table S5: Polymorphisms × sodium interactions influencing SBP detected from genome-wide analysis under dominant model

Variant	Chr	Position	CA	OA	Discovery				Replication				Combined			
					CGF	β_{GE}	SE(β_{GE})	*P*	CAF	β_{GE}	SE(β_{GE})	*P*	CAF	β_{GE}	SE(β_{GE})	*P*
rs1146341	1	118,983,663	G	A	0.945	-0.132	0.028	7.0E-06	0.955	0.022	0.038	5.6E-01	0.950	-0.066	0.022	2.7E-03
rs1907996	4	4,831,744	T	C	0.916	-0.124	0.023	2.1E-07	0.913	-0.022	0.025	3.8E-01	0.915	-0.071	0.017	2.2E-05
rs7668879	4	111,946,732	C	T	0.982	-0.225	0.048	6.5E-06	0.978	0.065	0.056	2.4E-01	0.980	-0.098	0.036	7.1E-03
rs10280905	7	41,649,220	A	T	0.674	0.068	0.014	5.5E-06	0.676	-0.006	0.015	6.9E-01	0.674	0.031	0.010	2.2E-03
rs258999	7	110,550,541	T	A	0.954	0.158	0.033	4.3E-06	0.963	-0.054	0.038	1.5E-01	0.958	0.061	0.024	1.1E-02
rs2616148	8	20,679,658	G	T	0.949	0.141	0.029	3.6E-06	0.939	0.014	0.027	5.9E-01	0.944	0.066	0.019	6.4E-04
rs58325975	9	126,175,233	T	C	0.833	0.087	0.018	4.9E-06	0.831	0.032	0.019	8.3E-02	0.832	0.056	0.013	1.2E-05
rs10835507	11	29,312,419	G	A	0.946	0.137	0.030	9.9E-06	0.949	0.006	0.035	8.7E-01	0.948	0.079	0.022	4.6E-04
rs11223575	11	100,171,483	A	G	0.899	-0.104	0.022	3.7E-06	0.890	-0.009	0.022	6.9E-01	0.895	-0.056	0.015	3.0E-04
rs10891748	11	114,642,268	A	T	0.847	-0.088	0.019	7.0E-06	0.838	-0.026	0.019	1.7E-01	0.843	-0.057	0.013	1.7E-05
rs2331495	14	22,586,518	C	T	0.429	-0.068	0.014	1.3E-06	0.469	-0.021	0.014	1.4E-01	0.448	-0.041	0.010	1.7E-05
rs9912179	17	77,415,753	C	A	0.769	-0.075	0.016	5.8E-06	0.777	0.004	0.017	8.1E-01	0.773	-0.034	0.011	2.6E-03
rs10401165	19	38,671,201	A	G	0.988	0.338	0.068	1.7E-06	0.990	-0.064	0.081	4.3E-01	0.989	0.171	0.052	1.0E-03

Chr, chromosome; CA, coded allele; OA, other allele; CGF, coded genotype frequency (*i.e.*, frequency of coded allele carriers)

Table S6: Polymorphisms × sodium interactions influencing SBP detected from genome-wide analysis under recessive model

Variant	Chr	Position	CA	OA	Discovery				Replication				Combined			
					CGF	β_{GE}	SE(β_{GE})	*P*	CAF	β_{GE}	SE(β_{GE})	*P*	CAF	β_{GE}	SE(β_{GE})	*P*
rs2470551	3	15,657,893	C	T	0.524	-0.064	0.013	6.4E-06	0.519	-0.006	0.014	6.9E-01	0.522	-0.035	0.010	2.2E-04
rs12506029	4	56,534,709	A	G	0.303	0.069	0.015	7.8E-06	0.290	-0.031	0.015	4.5E-02	0.297	0.017	0.011	9.7E-02
rs74804235	4	147,780,487	T	C	0.843	-0.087	0.018	5.4E-06	0.858	-0.031	0.021	1.4E-01	0.850	-0.060	0.014	9.8E-06
rs79699215	4	152,352,727	T	C	0.973	0.176	0.037	5.8E-06	0.977	-0.011	0.047	8.2E-01	0.975	0.103	0.029	4.5E-04
rs199624187	4	161,353,109	AT	A	0.869	-0.100	0.019	1.0E-06	0.871	-0.006	0.022	7.9E-01	0.870	-0.054	0.014	1.7E-04
rs146408813	5	154,125,572	A	G	0.930	-0.126	0.027	9.8E-06	0.921	-0.022	0.026	4.1E-01	0.926	-0.070	0.019	2.0E-04
rs2259056	8	20,663,677	A	T	0.085	-0.119	0.024	2.6E-06	0.109	-0.003	0.022	9.0E-01	0.097	-0.048	0.016	2.4E-03
rs182765532	13	62,124,175	T	C	0.936	-0.132	0.027	3.4E-06	0.929	-0.019	0.027	4.7E-01	0.933	-0.068	0.019	3.0E-04
rs8022678	14	99,366,690	G	A	0.520	-0.064	0.013	4.7E-06	0.549	-0.049	0.014	4.1E-04	0.534	-0.056	0.010	4.3E-09
rs6540086	16	87,853,820	T	G	0.151	0.085	0.018	8.2E-06	0.151	0.001	0.019	9.7E-01	0.151	0.045	0.013	5.0E-04
rs594517	18	76,062,527	A	G	0.634	-0.066	0.014	4.8E-06	0.607	0.002	0.014	9.0E-01	0.621	-0.032	0.010	1.0E-03
19:8137604G>GCACACACA	19	8,137,604	G	GCACACACA	0.732	-0.075	0.015	2.7E-06	0.726	0.007	0.016	6.5E-01	0.729	-0.033	0.011	2.3E-03
rs8125262	20	18,761,682	G	C	0.749	0.072	0.015	5.8E-06	0.757	-0.013	0.016	4.2E-01	0.753	0.030	0.011	6.3E-03

Chr, chromosome; CA, coded allele; OA, other allele; CGF, coded genotype frequency (*i.e.*, frequency of coded allele homo subjects)

Table S7: Polymorphisms × sodium interactions influencing DBP detected from genome-wide analysis under dosage model

Variant	Chr	Position	CA	OA	Discovery				Replication				Combined			
					CAF	β_{GE}	SE(β_{GE})	*P*	CAF	β_{GE}	SE(β_{GE})	*P*	CAF	β_{GE}	SE(β_{GE})	*P*
rs145889214	1	201,816,692	A	G	0.987	-0.130	0.027	3.0E-06	0.980	0.007	0.022	7.4E-01	0.984	-0.048	0.017	4.7E-03
rs144892325	3	56,388,527	C	A	0.985	0.130	0.028	8.4E-06	0.979	-0.019	0.021	3.8E-01	0.982	0.039	0.017	2.3E-02
rs751348	5	168,710,623	C	T	0.869	0.041	0.009	8.3E-06	0.857	-0.011	0.009	2.0E-01	0.863	0.016	0.006	7.1E-03
rs111573571	6	2,811,803	A	G	0.756	-0.035	0.008	9.5E-06	0.770	-0.005	0.007	4.8E-01	0.763	-0.019	0.005	2.9E-04
7:107316019CTT>C	7	107,316,019	CTT	C	0.869	-0.049	0.009	5.9E-07	0.874	0.017	0.009	7.6E-02	0.872	-0.018	0.007	6.5E-03
rs57898979	8	13,838,931	T	C	0.879	-0.044	0.009	6.3E-06	0.872	-0.001	0.009	8.9E-01	0.876	-0.021	0.006	7.0E-04
rs199578376	8	20,665,333	A	AT	0.767	0.035	0.007	5.6E-06	0.771	0.006	0.007	3.6E-01	0.769	0.019	0.005	2.0E-04
rs147635494	10	115,656,027	A	G	0.968	-0.091	0.020	7.2E-06	0.971	-0.012	0.018	5.0E-01	0.970	-0.046	0.013	3.9E-04
rs2103985	20	18,755,611	G	A	0.863	0.041	0.008	2.6E-06	0.869	-0.001	0.009	9.2E-01	0.866	0.021	0.006	3.8E-04
rs6114419	20	24,019,385	A	G	0.812	0.038	0.008	1.5E-06	0.806	0.001	0.007	9.0E-01	0.809	0.019	0.005	2.7E-04

Chr, chromosome; CA, coded allele; OA, other allele; CAF, coded allele frequency

Table S8: Polymorphisms × sodium interactions influencing DBP detected from genome-wide analysis under additive model

Variant	Chr	Position	CA	OA	Discovery				Replication				Combined			
					CAF	β_{GE}	SE(β_{GE})	*P*	CAF	β_{GE}	SE(β_{GE})	*P*	CAF	β_{GE}	SE(β_{GE})	*P*
rs1146341	1	118,983,663	G	A	0.776	-0.033	0.007	6.8E-06	0.795	0.015	0.007	4.0E-02	0.785	-0.009	0.005	6.0E-02
rs7561020	2	162,997,265	G	A	0.420	0.028	0.006	8.3E-06	0.433	-0.004	0.006	5.0E-01	0.426	0.013	0.004	2.9E-03
rs144892325	3	56,388,527	C	A	0.985	0.132	0.027	1.9E-06	0.979	-0.014	0.020	4.8E-01	0.982	0.041	0.016	1.0E-02
rs751348	5	168,710,623	C	T	0.869	0.041	0.009	8.3E-06	0.857	-0.011	0.009	2.0E-01	0.863	0.016	0.006	7.1E-03
7:52774883G>A	7	52,774,883	G	A	0.985	-0.110	0.024	8.4E-06	0.982	0.026	0.024	2.9E-01	0.983	-0.042	0.017	1.2E-02
7:107316019CTT>C	7	107,316,019	CTT	C	0.867	-0.041	0.009	4.6E-06	0.872	0.014	0.009	9.7E-02	0.869	-0.015	0.006	1.1E-02
rs7776974	7	131,947,817	G	A	0.835	-0.038	0.008	7.9E-06	0.832	-0.006	0.008	4.6E-01	0.833	-0.020	0.006	4.3E-04
rs62476815	7	157,439,855	C	T	0.840	0.040	0.009	5.6E-06	0.831	0.007	0.008	3.5E-01	0.836	0.021	0.006	1.9E-04
rs57898979	8	13,838,931	T	C	0.877	-0.043	0.009	4.2E-06	0.871	-0.002	0.008	8.5E-01	0.874	-0.021	0.006	4.9E-04
rs2119699	8	20,664,812	C	T	0.770	0.034	0.007	5.6E-06	0.774	0.007	0.007	3.0E-01	0.772	0.019	0.005	1.2E-04
rs345188	17	58,230,946	A	G	0.957	0.068	0.014	5.5E-06	0.961	-0.003	0.015	8.2E-01	0.959	0.034	0.010	9.3E-04
rs7253456	19	2,098,015	T	C	0.772	-0.033	0.007	9.6E-06	0.776	-0.013	0.007	5.6E-02	0.774	-0.023	0.005	5.3E-06
rs8125262	20	18,761,682	G	C	0.863	0.041	0.008	2.8E-06	0.870	-0.001	0.009	9.5E-01	0.867	0.021	0.006	3.4E-04
rs6049351	20	24,017,101	G	T	0.812	0.038	0.008	1.5E-06	0.806	0.001	0.007	9.0E-01	0.809	0.019	0.005	2.7E-04

Chr, chromosome; CA, coded allele; OA, other allele; CAF, coded allele frequency

Table S9: Polymorphisms × sodium interactions influencing DBP detected from genome-wide analysis under dominant model

Variant	Chr	Position	CA	OA	Discovery				Replication				Combined			
					CGF	β_{GE}	SE(β_{GE})	*P*	CAF	β_{GE}	SE(β_{GE})	*P*	CAF	β_{GE}	SE(β_{GE})	*P*
rs6669918	1	26,427,055	C	A	0.964	-0.119	0.025	2.5E-06	0.971	-0.005	0.026	8.4E-01	0.967	-0.059	0.018	8.8E-04
rs6425203	1	173,064,279	T	C	0.967	-0.132	0.028	4.4E-06	0.954	0.011	0.022	6.1E-01	0.961	-0.048	0.017	5.4E-03
rs60384468	3	5,419,510	C	A	0.944	-0.083	0.018	5.5E-06	0.941	0.013	0.018	4.7E-01	0.943	-0.036	0.013	4.6E-03
rs12655979	5	9,317,252	T	C	0.980	0.166	0.034	1.4E-06	0.978	0.046	0.027	8.6E-02	0.979	0.093	0.021	6.9E-06
rs2616191	8	20,669,205	G	T	0.952	0.111	0.019	1.5E-08	0.941	0.019	0.016	2.4E-01	0.947	0.056	0.012	4.9E-06
rs13257008	8	25,649,192	C	A	0.963	-0.105	0.023	7.8E-06	0.965	-0.016	0.021	4.6E-01	0.964	-0.056	0.015	2.7E-04
rs57873233	9	86,018,500	C	T	0.988	-0.193	0.042	8.9E-06	0.988	0.022	0.054	6.8E-01	0.988	-0.109	0.033	7.9E-04
rs11223575	11	100,171,483	A	G	0.899	-0.065	0.014	3.4E-06	0.890	-0.009	0.013	4.8E-01	0.895	-0.036	0.009	1.3E-04
rs76228738	12	54,442,915	T	C	0.977	-0.120	0.026	5.7E-06	0.974	-0.033	0.023	1.6E-01	0.976	-0.070	0.017	4.6E-05
rs78288559	14	82,576,506	A	G	0.912	-0.080	0.017	6.0E-06	0.912	0.008	0.016	6.0E-01	0.912	-0.034	0.012	3.7E-03
rs1351544	15	61,042,867	G	T	0.964	-0.109	0.023	4.1E-06	0.956	0.017	0.022	4.2E-01	0.960	-0.042	0.016	7.0E-03
rs34720531	20	7,139,186	AT	A	0.950	0.097	0.020	3.6E-06	0.962	-0.022	0.021	2.8E-01	0.956	0.036	0.014	1.2E-02
rs149408729	22	33,176,871	T	TTGCC	0.980	-0.136	0.029	5.1E-06	0.978	0.005	0.030	8.8E-01	0.979	-0.071	0.021	6.3E-04

Chr, chromosome; CA, coded allele; OA, other allele; CGF, coded genotype frequency (*i.e.*, frequency of coded allele carriers)

Table S10: Polymorphisms × sodium interactions influencing DBP detected from genome-wide analysis under recessive model

Variant	Chr	Position	CA	OA	Discovery				Replication				Combined			
					CGF	β_{GE}	SE(β_{GE})	*P*	CAF	β_{GE}	SE(β_{GE})	*P*	CAF	β_{GE}	SE(β_{GE})	*P*
rs144892325	3	56,388,527	C	A	0.970	0.131	0.027	2.0E-06	0.959	-0.015	0.021	4.8E-01	0.965	0.043	0.016	8.6E-03
rs12523280	5	28,433,239	C	T	0.412	0.040	0.009	9.0E-06	0.438	0.008	0.008	3.5E-01	0.425	0.023	0.006	1.2E-04
7:52774883G>A	7	52,774,883	G	A	0.969	-0.110	0.024	8.9E-06	0.964	0.026	0.025	2.8E-01	0.967	-0.042	0.017	1.2E-02
rs62476815	7	157,439,855	C	T	0.706	0.047	0.009	2.1E-06	0.692	0.008	0.009	3.7E-01	0.699	0.026	0.006	6.4E-05
rs2259056	8	20,663,677	A	T	0.085	-0.083	0.015	2.1E-07	0.109	-0.009	0.013	4.5E-01	0.097	-0.036	0.010	2.1E-04
rs7025549	9	16,881,256	T	G	0.446	0.041	0.009	5.4E-06	0.469	-0.003	0.008	7.5E-01	0.457	0.019	0.006	1.4E-03
rs345188	17	58,230,946	A	G	0.916	0.070	0.015	7.5E-06	0.924	-0.007	0.016	6.7E-01	0.920	0.035	0.011	1.2E-03
rs45458192	19	19,750,822	C	T	0.471	-0.040	0.009	8.6E-06	0.441	-0.002	0.008	7.8E-01	0.456	-0.021	0.006	4.7E-04
rs1321323	20	18,765,007	T	C	0.758	0.051	0.010	8.1E-07	0.769	0.000	0.010	1.0E+00	0.763	0.025	0.007	3.3E-04

Chr, chromosome; CA, coded allele; OA, other allele; CGF, coded genotype frequency (*i.e.*, frequency of coded allele homo subjects)

Table S11. Characteristics of study populations stratified by rs8022678 genotype

Population	Variable	rs8022678		*P**
		GG homo	A carrier	
Discovery	*N*	2,353	2,174	–
	Female, %	65.3	67.6	0.10
	Age, year (mean ± SD)	58.9 ± 11.8	59.1 ± 11.8	0.47
	SBP, mm Hg (mean ± SD)	128.4 ± 19.3	128.0 ± 19.2	0.35
	DBP, mm Hg (mean ± SD)	77.0 ± 11.8	76.4 ± 12.0	0.16
	Hypertension, %	38.9	36.9	0.17
	Hypertension medication, %	24.7	22.6	0.10
	BMI, kg/m^2 (mean ± SD)	23.6 ± 3.6	23.5 ± 3.6	0.20
	Daily sodium consumption, mEq/day (mean ± SD)	164.4 ± 37.5	165.3 ± 38.4	0.77
Replication	*N*	2,328	1,913	–
	Female, %	63.5	63.7	0.93
	Age, year (mean ± SD)	62.4 ± 10.1	62.8 ± 9.6	0.42
	SBP, mm Hg (mean ± SD)	131.4 ± 18.8	131.6 ± 19.4	0.93
	DBP, mm Hg (mean ± SD)	76.3 ± 11.0	76.5 ± 11.0	0.66
	Hypertension, %	41.1	43.3	0.15
	Hypertension medication, %	23.7	23.9	0.87
	BMI, kg/m^2 (mean ± SD)	23.5 ± 3.4	23.5 ± 3.5	0.58
	Daily sodium consumption, mEq/day (mean ± SD)	176.8 ± 38.3	176.4 ± 38.7	0.62

SBP, systolic blood pressure; DBP, diastolic blood pressure; BMI, body mass index; SD, standard deviation

*Significance was tested by Wilcoxon rank sum test

Table S12. Sodium effect on blood pressure stratified by rs8022678 genotype in discovery cohort

rs8022678	Variable	Tertile of daily sodium consumption		
		T1 (Low)	T2 (Medium)	T3 (High)
All	Range, mEq/day (mg/day)	<147.5 (<3,393)	147.5 – 178.0 (3,393–4,094)	>178.0 (>4,094)
	SBP (mean ± SD), mm Hg	124.7 ± 16.1	125.4 ± 15.9	127.4 ± 17.0
	Mean difference in SBP, mm Hg	ref.	0.7	2.7
	P	ref.	1.9E-01	3.5E-05
	DBP (mean ± SD), mm Hg	75.0 ± 10.5	75.4 ± 10.3	76.2 ± 11.0
	Mean difference in DBP, mm Hg	ref.	0.4	1.2
	P	ref.	4.0E-01	8.1E-03
GG homo	SBP (mean ± SD), mm Hg	126.5 ± 16.4	124.9 ± 16.0	126.5 ± 17.0
	Mean difference in SBP, mm Hg	ref.	-1.6	0.0
	P	ref.	5.3E-02	4.9E-01
	DBP (mean ± SD), mm Hg	76.1 ± 10.6	75.2 ± 10.2	75.8 ± 10.9
	Mean difference in DBP, mm Hg	ref.	-0.9	-0.2
	P	ref.	5.0E-02	3.5E-01
A carrier	SBP (mean ± SD), mm Hg	122.8 ± 15.6	126.0 ± 15.8	128.4 ± 17.0
	Mean difference in SBP, mm Hg	ref.	3.2	5.5
	P	ref.	7.2E-05	1.3E-11
	DBP (mean ± SD), mm Hg	73.9 ± 10.3	75.6 ± 10.3	76.6 ± 11.2
	Mean difference in DBP, mm Hg	ref.	1.7	2.7
	P	ref.	1.2E-03	2.0E-06

We analyzed measured SBP and DBP rather than imputed SBP and DBP

Table S13. Sodium effect on blood pressure stratified by rs8022678 genotype in replication cohort

rs8022678	Variable	Tertile of daily sodium consumption		
		T1 (Low)	T2 (Medium)	T3 (High)
All	Range, mEq/day	<159.9	159.9 – 191.7	>191.7
	(mg/day)	(<3,678)	(3,678–4,409)	(>4,409)
	SBP (mean ± SD), mm Hg	126.4 ± 17.0	129.3 ± 16.6	131.6 ± 16.7
	Mean difference in SBP, mm Hg	ref.	2.9	5.1
	P	ref.	3.7E-06	4.5E-15
	DBP (mean ± SD), mm Hg	74.0 ± 10.1	75.3 ± 9.9	76.3 ± 10.1
	Mean difference in DBP, mm Hg	ref.	1.3	2.3
	P	ref.	7.7E-04	1.7E-09
GG homo	SBP (mean ± SD), mm Hg	127.2 ± 17.0	129.4 ± 16.0	130.8 ± 16.5
	Mean difference in SBP, mm Hg	ref.	2.3	3.6
	P	ref.	3.0E-03	1.3E-05
	DBP (mean ± SD), mm Hg	74.2 ± 10.1	75.0 ± 9.8	76.1 ± 10.1
	Mean difference in DBP, mm Hg	ref.	0.8	1.9
	P	ref.	1.3E-01	3.6E-04
A carrier	SBP (mean ± SD), mm Hg	125.5 ± 17.1	129.2 ± 17.2	132.5 ± 17.0
	Mean difference in SBP, mm Hg	ref.	3.7	7.0
	P	ref.	3.2E-04	7.5E-12
	DBP (mean ± SD), mm Hg	73.7 ± 10.1	75.6 ± 10.0	76.6 ± 10.0
	Mean difference in DBP, mm Hg	ref.	1.9	2.9
	P	ref.	8.1E-04	4.6E-07

We analyzed measured SBP and DBP rather than imputed SBP and DBP

Table S14. Sodium effect on blood pressure stratified by rs8022678 genotype in discovery cohort with excluding subjects taking antihypertensive medication

rs8022678	Variable	Tertile of daily sodium consumption		
		T1 (Low)	T2 (Medium)	T3 (High)
All	Range, mEq/day (mg/day)	<145.6 (<3,349)	145.6 – 175.3 (3,349–4,032)	>175.3 (>4,032)
	SBP (mean ± SD), mm Hg	122.8 ± 16.0	122.9 ± 15.9	125.2 ± 16.6
	Mean difference in SBP, mm Hg	ref.	0.1	2.4
	P	ref.	7.4E-01	5.7E-04
	DBP (mean ± SD), mm Hg	74.2 ± 10.3	74.0 ± 10.0	75.3 ± 11.0
	Mean difference in DBP, mm Hg	ref.	-0.2	1.1
	P	ref.	5.9E-01	2.9E-02
GG homo	SBP (mean ± SD), mm Hg	124.3 ± 16.6	122.7 ± 16.1	124.0 ± 16.4
	Mean difference in SBP, mm Hg	ref.	-1.6	-0.3
	P	ref.	1.3E-01	4.5E-01
	DBP (mean ± SD), mm Hg	75.2 ± 10.4	73.8 ± 10.2	74.9 ± 10.8
	Mean difference in DBP, mm Hg	ref.	-1.3	-0.3
	P	ref.	1.4E-02	4.3E-01
A carrier	SBP (mean ± SD), mm Hg	121.2 ± 15.3	123.1 ± 15.7	126.7 ± 16.7
	Mean difference in SBP, mm Hg	ref.	1.9	5.4
	P	ref.	3.2E-02	3.9E-09
	DBP (mean ± SD), mm Hg	73.2 ± 10.2	74.1 ± 9.9	75.7 ± 11.2
	Mean difference in DBP, mm Hg	ref.	0.9	2.6
	P	ref.	7.7E-02	9.9E-05

We analyzed measured SBP and DBP rather than imputed SBP and DBP. Note that measured and imputed BPs were exactly same for all subjects included in the analysis for this Table.

Table S15. Sodium effect on blood pressure stratified by rs8022678 genotype in replication cohort with excluding subjects taking antihypertensive medication

rs8022678	Variable	Tertile of daily sodium consumption		
		T1 (Low)	T2 (Medium)	T3 (High)
All	Range, mEq/day	<160.0	160.0 – 191.3	>191.3
	(mg/day)	(<3,680)	(3,680–4,400)	(>4,400)
	SBP (mean ± SD), mm Hg	125.3 ± 17.1	127.4 ± 16.5	129.4 ± 16.8
	Mean difference in SBP, mm Hg	ref.	2.1	4.2
	P	ref.	2.6E-03	3.8E-08
	DBP (mean ± SD), mm Hg	73.6 ± 10.2	74.7 ± 10.1	75.5 ± 10.0
	Mean difference in DBP, mm Hg	ref.	1.1	1.9
	P	ref.	1.3E-02	1.8E-05
GG homo	SBP (mean ± SD), mm Hg	125.8 ± 16.8	127.4 ± 16.1	129.0 ± 16.9
	Mean difference in SBP, mm Hg	ref.	1.6	3.2
	P	ref.	4.7E-02	7.2E-04
	DBP (mean ± SD), mm Hg	73.7 ± 10.1	74.4 ± 10.0	75.5 ± 10.2
	Mean difference in DBP, mm Hg	ref.	0.7	1.7
	P	ref.	3.0E-01	4.3E-03
A carrier	SBP (mean ± SD), mm Hg	124.7 ± 17.5	127.3 ± 16.9	129.9 ± 16.6
	Mean difference in SBP, mm Hg	ref.	2.7	5.3
	P	ref.	2.2E-02	8.0E-06
	DBP (mean ± SD), mm Hg	73.4 ± 10.3	75.1 ± 10.2	75.4 ± 9.9
	Mean difference in DBP, mm Hg	ref.	1.7	2.0
	P	ref.	1.1E-02	1.2E-03

We analyzed measured SBP and DBP rather than imputed SBP and DBP. Note that measured and imputed BPs were exactly same for all subjects included in the analysis for this Table.

Table S16. Sodium effect on blood pressure stratified by rs8022678 genotype in discovery cohort with excluding hypertensive subjects

rs8022678	Variable	Tertile of daily sodium consumption		
		T1 (Low)	T2 (Medium)	T3 (High)
All	Range, mEq/day	<144.0	144.0 – 173.7	>173.7
	(mg/day)	(<3,312)	(3,312–3,995)	(>3,995)
	SBP (mean ± SD), mm Hg	117.0 ± 12.5	117.5 ± 12.1	118.9 ± 11.4
	Mean difference in SBP, mm Hg	ref.	0.5	1.9
	P	ref.	3.4E-01	4.4E-04
	DBP (mean ± SD), mm Hg	71.2 ± 8.8	71.0 ± 8.3	71.6 ± 8.3
	Mean difference in DBP, mm Hg	ref.	-0.2	0.4
	P	ref.	5.8E-01	3.2E-01
GG homo	SBP (mean ± SD), mm Hg	118.1 ± 12.9	117.5 ± 12.9	117.9 ± 11.2
	Mean difference in SBP, mm Hg	ref.	-0.6	-0.2
	P	ref.	4.5E-01	7.5E-01
	DBP (mean ± SD), mm Hg	72.1 ± 8.7	70.8 ± 8.4	71.4 ± 8.3
	Mean difference in DBP, mm Hg	ref.	-1.3	-0.7
	P	ref.	9.9E-03	1.6E-01
A carrier	SBP (mean ± SD), mm Hg	115.9 ± 11.9	117.6 ± 11.4	120.0 ± 11.5
	Mean difference in SBP, mm Hg	ref.	1.7	4.1
	P	ref.	2.0E-02	3.1E-08
	DBP (mean ± SD), mm Hg	70.3 ± 8.8	71.2 ± 8.3	71.8 ± 8.4
	Mean difference in DBP, mm Hg	ref.	0.9	1.5
	P	ref.	6.6E-02	6.5E-03

We analyzed measured SBP and DBP rather than imputed SBP and DBP. Note that measured and imputed BPs were exactly same for all subjects included in the analysis for this Table.

Table S17. Sodium effect on blood pressure stratified by rs8022678 genotype in replication cohort with excluding hypertensive subjects

rs8022678	Variable	Tertile of daily sodium consumption		
		T1 (Low)	T2 (Medium)	T3 (High)
All	Range, mEq/day	<158.2	158.2 – 188.9	>188.9
	(mg/day)	(<3,639)	(3,639–4,345)	(>4,345)
	SBP (mean ± SD), mm Hg	118.4 ± 12.1	120.2 ± 11.4	120.7 ± 10.8
	Mean difference in SBP, mm Hg	ref.	1.8	2.3
	P	ref.	5.1E-03	5.4E-04
	DBP (mean ± SD), mm Hg	70.4 ± 8.2	71.0 ± 7.8	71.2 ± 7.6
	Mean difference in DBP, mm Hg	ref.	0.7	0.9
	P	ref.	1.1E-01	2.4E-02
GG homo	SBP (mean ± SD), mm Hg	119.2 ± 11.6	120.6 ± 11.3	120.7 ± 11.1
	Mean difference in SBP, mm Hg	ref.	1.4	1.4
	P	ref.	6.4E-02	7.1E-02
	DBP (mean ± SD), mm Hg	70.7 ± 8.2	70.8 ± 7.9	71.2 ± 7.7
	Mean difference in DBP, mm Hg	ref.	0.2	0.5
	P	ref.	8.3E-01	3.5E-01
A carrier	SBP (mean ± SD), mm Hg	117.5 ± 12.6	119.7 ± 11.5	120.8 ± 10.5
	Mean difference in SBP, mm Hg	ref.	2.2	3.3
	P	ref.	3.1E-02	1.4E-03
	DBP (mean ± SD), mm Hg	70.0 ± 8.3	71.2 ± 7.8	71.3 ± 7.6
	Mean difference in DBP, mm Hg	ref.	1.3	1.3
	P	ref.	3.7E-02	1.8E-02

We analyzed measured SBP and DBP rather than imputed SBP and DBP. Note that measured and imputed BPs were exactly same for all subjects included in the analysis for this Table.

Table S18. Comparison of the probability to be hypertension between rs8022678 A carriers versus rs8022678 A non-carriers

	Tertile of daily sodium consumption		
	T1 (Low)	T2 (Medium)	T3 (High)
Range, mEq/day	<153.1	153.1 – 185.2	>185.2
(mg/day)	(<3,521)	(3,521–4,260)	(>4,260)
Odds ratio	0.80	1.05	1.14
(95% confidence interval)	(0.68–0.94)	(0.89–1.22)	(0.98–1.32)
P	0.006	0.57	0.099

This association analysis was based on the combined dataset of the discovery and replication cohorts and was performed with a logistic regression model with adjustment for age, sex and cohort. The subgroups of rs8022678 A non-carriers was considered as reference.
